# Atomic layer deposition of nickel sulfide thin films and their thermal and electrochemical stability†

**DOI:** 10.1039/d5ta00663e

**Published:** 2025-07-10

**Authors:** Miika Mattinen, Johanna Schröder, Timo Hatanpää, Georgi Popov, Kenichiro Mizohata, Markku Leskelä, Thomas F. Jaramillo, Michaela Burke Stevens, Stacey F. Bent, Mikko Ritala

**Affiliations:** a Department of Chemistry, University of Helsinki P. O. Box 55 FI-00014 Finland miika.mattinen@helsinki.fi mikko.ritala@helsinki.fi; b Department of Chemical Engineering, Stanford University 443 Via Ortega Stanford California 94305 USA; c SUNCAT Center for Interface Science and Catalysis, SLAC National Accelerator Laboratory 2575 Sand Hill Road Menlo Park California 94025 USA; d Division of Materials Physics, Department of Physics, University of Helsinki P. O. Box 43 FI-00014 Finland; e Department of Energy Science and Engineering, Stanford University 443 Via Ortega Stanford California 94305 USA

## Abstract

Nickel sulfides (NiS_*x*_) show promise for a range of energy and other applications, but their (in)stability under processing and operating conditions is scarcely studied. Herein, we have developed a new NiS_*x*_ atomic layer deposition process using an easily synthesized NiCl_2_(TMPDA) precursor (TMPDA = *N*,*N*,*N*′,*N*′-tetramethyl-1,3-propanediamine) with H_2_S. Thin films deposited at 165–225 °C consist mostly of the β-NiS phase and display low resistivity (∼40–120 μΩ cm), high purity (<3 at% impurities), and a rough morphology. The thermal stability of the NiS_*x*_ thin films is studied using high-temperature X-ray diffraction, revealing that structural and compositional changes occur in reducing, inert, and oxidizing atmospheres at approximately 300–400 °C. Under electrochemical water splitting conditions, the films are unstable in acid due to dissolution, especially at oxidizing potentials. In an alkaline electrolyte, we do not observe Ni dissolution, but β-NiS transforms to Ni_3_S_2_ under HER conditions, possibly supplemented with Ni and/or Ni(OH)_2_ species. Under alkaline OER, all sulfur is lost and NiOOH is formed. In addition to offering an attractive, scalable route to the synthesis of NiS_*x*_ thin films, our work highlights the importance of thermal and electrochemical (in)stability of sulfides as a crucial step for understanding and engineering materials for energy and other applications.

## Abstract 2

An atomic layer deposition process for NiS_*x*_ thin films is developed. The deposited β-NiS films undergo structural and compositional changes during water splitting and high-temperature annealing.

## Graphical abstract

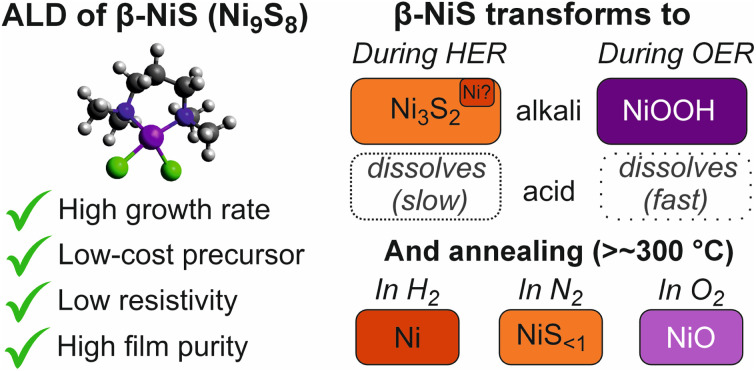

## Introduction

Nickel sulfides (NiS_*x*_) form a diverse group of materials with different compositions and crystal structures, including five that are observed as minerals: rhombohedral Ni_3_S_2_, orthorhombic Ni_9_S_8_, rhombohedral β-NiS, cubic Ni_3_S_4_, and cubic NiS_2_ as well as high-temperature phases orthorhombic Ni_7_S_6_ and hexagonal α-NiS.^1,2^ Most of these phases are highly conductive metallic or degenerate semiconducting materials.^3–12^ Their electronic properties together with the low toxicity and earth abundance of nickel and sulfur make NiS_*x*_ promising electrode materials for a range of energy applications, including supercapacitors,^13,14^ lithium-ion batteries,^15–17^ and dye-sensitized solar cells,^5,7,18^ as well as electrochemical^6,19–23^ and photoelectrochemical^14^ water splitting.

The stability of NiS_*x*_ under processing and operating conditions encountered in different applications has received relatively little attention. The processing of devices from microelectronics to solar cells and beyond often includes annealing steps, which can lead to changes in film composition and structure. In operation of electrocatalysts, the electric potential together with the presence of the electrolyte challenge the stability of the catalyst. Nickel sulfides are regarded as promising water splitting electrocatalysts.^6,19–25^ However, Pourbaix diagrams predict NiS_*x*_ to be unstable under typical operating conditions of both the reducing (*i.e.* hydrogen evolution reaction, HER) and oxidizing (*i.e.* oxygen evolution reaction, OER) half-reactions of water splitting.^25–27^ Commercial electrolyzer technologies operate under alkaline (alkaline electrolyzers^28,29^ and anion exchange membrane (AEM) electrolyzers^28,30^) or acidic conditions (proton exchange membrane (PEM) electrolyzers^31^). While numerous studies have reported NiS_*x*_ to be active for both the HER and OER, investigations into the stability and the actual catalytic species remain rather scarce. Under alkaline OER conditions, NiS_*x*_ has been reported to transform to NiOOH prior to the OER, making NiS_*x*_ a precatalyst to the actual NiOOH catalyst.^22,25,32–34^ Regardless, consensus on the rate and extent (depth) of this transformation has not been reached.^25^ For the less harsh HER conditions, it remains common to assume NiS_*x*_ to be stable. However, recent reports have suggested that NiS_*x*_ may transform to either another NiS_*x*_ phase,^35,36^ Ni metal,^37^ NiO_*x*_S_*y*_,^38^ or Ni(OH)_2_ (ref. 39 and 40) under alkaline HER conditions. A recent review by Kawashima *et al.*^41^ suggested that the majority of metal chalcogenides, including NiS_*x*_, may undergo some degree of structural and/or compositional change under alkaline HER conditions. For the HER in acid, good stability appears to be the prevailing view, yet dissolution of NiS_*x*_ has also been observed.^19,42^ For both the OER and HER, there is limited understanding of the effects of NiS_*x*_ compositions as well as the preparation method. Thus, questions on the stability of NiS_*x*_ under water splitting conditions and the identity of the actual catalytic species remain largely unanswered.

A variety of methods have been used to deposit NiS_*x*_ thin films and nanoparticles, including solid-state,^17^ hydrothermal,^23^ solvothermal,^13,21^ and colloidal synthesis,^15^ electrodeposition,^18^ sulfurization,^19,20^ chemical vapor deposition (CVD),^43^ pulsed laser deposition,^44^ and molecular beam epitaxy.^45^ Nevertheless, existing methods often fail to meet one or more of the following desirable aspects: high purity and crystallinity, thickness control, scalability, uniform coating of three-dimensional substrates, and low processing temperatures. To overcome these challenges, we use atomic layer deposition (ALD), an advanced CVD technique relying on self-limiting surface reactions of alternately supplied precursors. ALD offers unmatched thickness control, reproducibility, scalability and the ability to coat both large and complexly shaped substrates with uniform layers of thin films or nanoparticles.^46–48^ The advantageous characteristics of ALD along with the rising interest in NiS_*x*_ have led to the development of several NiS_*x*_ ALD processes. β-NiS films have been deposited using β-diketonates Ni(thd)_2_ (ref. 5) and Ni(acac)_2_ (ref. 49) with H_2_S, while amorphous NiS (ref. 50) and crystalline Ni_9_S_8_ (ref. 22) have been reported using an amidinate Ni(^*t*^BuAMD)_2_ with H_2_S. Ni(^*t*^BuAMD)_2_ can also be used with di-*tert*-butyl-disulfide^51^ and H_2_S plasma^52^ to deposit crystalline Ni_9_S_8_ and NiS_2_ films, respectively. An aminoalkoxide, Ni(dmamb)_2_, combined with H_2_S has been reported to result in either crystalline Ni_3_S_2_ (ref. 6 and 7) or β-NiS (ref. 53) films. Despite several published processes, controlling the deposited NiS_*x*_ phase remains difficult, and none of the processes can deposit highly pure and conductive NiS_*x*_ films with a high growth rate from affordable precursors. Conductivity is key to its application as an electrode, while growth rate and precursor cost have a significant effect on the industrial scalability of the process. Recently, NiCl_2_(TMPDA) (TMPDA = *N*,*N*,*N*′,*N*′-tetramethyl-1,3-propanediamine) was introduced as a promising low-cost alternative to the existing nickel ALD precursors^54–56^ that provides sufficient volatility, thermal stability, and reactivity, but NiS_*x*_ has not yet been deposited using this precursor.

In this work, we used NiCl_2_(TMPDA) with H_2_S at 165–225 °C, yielding crystalline films consisting of mainly the β-NiS phase. The film properties including crystallinity, morphology, impurities, and electrical properties were assessed. We investigated the stability of the deposited films in reducing, inert, and oxidizing atmospheres at elevated temperatures to mimic conditions encountered during processing of various devices. We then evaluated β-NiS for both half reactions of electrochemical water splitting (HER and OER) under both alkaline and acidic conditions. Thin films are well suited for investigating stability, which is a crucial step in the process of engineering advanced electrodes for electrocatalysis and other applications.

## Results and discussion

### ALD process development

**Fig. 1:**
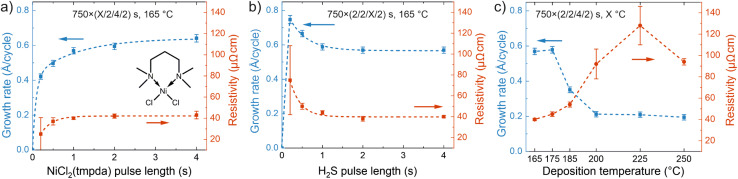
ALD process development. Growth rate and resistivity *versus* (a) NiCl_2_(TMPDA), (b) H_2_S pulse length, and (c) deposition temperature. The nickel precursor is shown as an inset in (a). Thicknesses were measured by energy-dispersive X-ray spectrometry (EDS). The growth rate error bars represent statistical measurement uncertainty (one standard deviation). The resistivity error bars represent variation in sheet resistance over the 5 × 5 cm^2^ substrate (one standard deviation, calculated using a single thickness value per sample). The films were deposited on silicon using, unless otherwise noted, 750 cycles with 2.0 s NiCl_2_(TMPDA) and 4.0 s H_2_S pulses, both followed by 2.0 s N_2_ purges, at 165 °C (denoted 750 × (2/2/4/2) s).

Nickel sulfide films were deposited by ALD using a new precursor combination of NiCl_2_(TMPDA) and H_2_S. The precursor pulses were separated by inert nitrogen (N_2_) purges and the NiCl_2_(TMPDA)–N_2_–H_2_S–N_2_ ALD cycle was repeated until the desired film thickness was reached. The growth characteristics were initially evaluated on silicon substrates at a temperature of 165 °C using 750 ALD cycles. With an increase of the NiCl_2_(TMPDA) pulse length, the growth rate leveled off by 1.0 s pulse time to approximately 0.6 Å per cycle (Fig. 1a), indicating the self-limiting precursor adsorption characteristic of ALD. A further increase of the pulse length from 1.0 to 4.0 s increased the growth rate slightly to approximately 0.7 Å per cycle, which we attribute to minor changes in phase composition, crystalline orientation, and morphology (see the section Effects of deposition temperature and film thickness on crystallinity, morphology, and composition and Note S3 in the ESI†). Pulsing only NiCl_2_(TMPDA) repeatedly resulted in negligible film growth, ruling out precursor decomposition (Fig. S1 in the ESI†). A low resistivity of 40–45 μΩ cm (using a four-point probe) and high uniformity (1–3% standard deviation of sheet resistance over 5 × 5 cm^2^ substrates) were obtained using a NiCl_2_(TMPDA) pulse length of at least 1.0 s. The films deposited on both Si and glass substrates had a shiny, metallic appearance.

When the H_2_S pulse length was increased from the shortest value of 0.2 s, the growth rate first decreased before it stabilized at 0.6 Å per cycle using H_2_S pulse lengths of at least 1.0 s (Fig. 1b). At the same time, the resistivity of the films decreased reaching approximately 40 μΩ cm at 2.0 s H_2_S pulse length. The film uniformity was good except for the shortest 0.2 s H_2_S pulse. The S/Ni ratio of the films remained unchanged at approximately 1.0 regardless of the pulse length (Fig. S2†). Regarding the purge steps, we observed that 1.0 s N_2_ purges after each precursor pulse were sufficient to remove unreacted precursors and byproducts (Fig. S3†). Based on these experiments, pulse lengths of 2.0 s for both NiCl_2_(TMPDA) and H_2_S and 2.0 s purges were chosen for further depositions to ensure operation in the saturated ALD regime.

As seen in Fig. 1c, the growth rate remained unchanged when the deposition temperature was increased from 165 to 175 °C, but a further increase led to a decrease in the growth rate to approximately 0.4 Å per cycle at 185 °C and 0.2 Å per cycle at 200–250 °C. Concurrently, the film resistivity increased from 40 μΩ cm at 165 °C to approximately 100 μΩ cm at 200–250 °C, and the non-uniformity estimated from the standard deviation of sheet resistance increased from <3% at 165 °C to ∼15% at 200–225 °C. The lowest deposition temperature of 165 °C was limited by the temperature of 157 °C required to reach a vapor pressure of 0.1 mbar for NiCl_2_(TMPDA), whereas visible decomposition of NiCl_2_(TMPDA) was observed at 250 °C, limiting the highest ALD temperature to 225 °C in accordance with the earlier studies of NiCl_2_(TMPDA).^55^ In summary, NiCl_2_(TMPDA) and H_2_S afford a well-behaved ALD process in the 165–225 °C temperature range.

### Effects of deposition temperature and film thickness on crystallinity, morphology, and composition

**Fig. 2:**
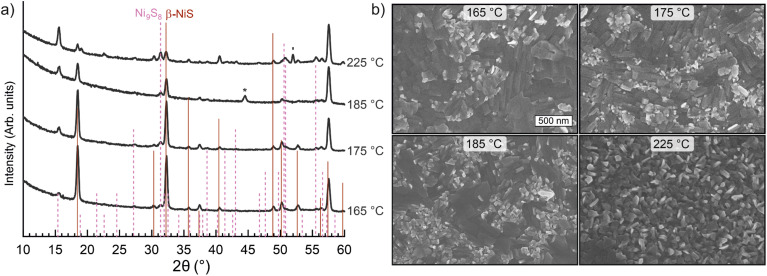
Characterization of films deposited at different temperatures (thickness 46–50 nm) using (a) grazing incidence XRD and (b) SEM. The films were deposited on silicon using 750 (165 °C, 175 °C), 1500 (185 °C), and 2250 cycles (225 °C). The position and height of the lines in (a) indicate peak positions and intensities of powder references (PDF 12-41 for β-NiS and 22-1193 for Ni_9_S_8_). Peaks from the sample stage of the XRD instrument and Si substrate are denoted with an asterisk and apostrophe.

Next, the morphology and crystallinity of the films deposited on silicon at different temperatures were studied. Since the deposition temperature affects the growth rate, the number of ALD cycles was adjusted to reach a similar thickness (44–48 nm). At 165 °C, relatively large features ranging from approximately 100 to 300 nm in width were observed on the surface of the films by scanning electron microscopy (SEM, Fig. 2b). With increasing deposition temperature, smaller, more rod-like features became more prominent on the film surface in comparison to the flatter, larger structures dominant at 165 °C. X-ray diffraction (XRD, Fig. 2a) showed the films to consist primarily of the rhombohedral β-NiS phase (millerite, powder diffraction file (PDF) 12-41) with a few additional peaks that were indexed to the orthorhombic Ni_9_S_8_ phase (godlevskite, PDF 22-1193).^57,58^ With increasing deposition temperature, the intensities of the Ni_9_S_8_ peaks increased and those of the β-NiS peaks decreased. The β-NiS phase was observed to have a preferred (300) orientation for all of the deposition temperatures, while the Ni_9_S_8_ phase became (111) textured at 225 °C and above, with no clear texture at lower temperatures (Note S2 and Fig. S7†). The changes in phase composition, preferred orientation, and morphology may at least partially explain the rather strong temperature-dependence of the growth rate (Fig. 1c). Raman spectra of the films deposited at 185–225 °C confirmed the presence of β-NiS (Fig. S5†). No other phases were detected, which may be due to their Raman inactivity (Note S1†).

The elemental composition of the films was analyzed by time-of-flight elastic recoil detection analysis (ToF-ERDA; Table S1†). The S/Ni ratio remained at 1.00–1.02 in all of the measured films deposited at 165–225 °C, in agreement with the main β-NiS phase. The films were highly pure, containing less than 2 at% of O, C, N, and H impurities in total that originated from the TMPDA ligands and the atmosphere. Accurate determination of the chlorine content was difficult due to its similar atomic mass with sulfur, but an upper limit of approximately 1 at% was estimated for the chlorine concentration. The high film purity indicates facile and complete surface reactions between NiCl_2_(TMPDA) and H_2_S. Thus, we conclude that pure, crystalline films consisting mainly of the β-NiS phase can be deposited within the temperature range of 165–225 °C. At the higher end of the temperature range, the contribution of the additional Ni_9_S_8_ phase increases and the size of surface features decreases.

**Fig. 3:**
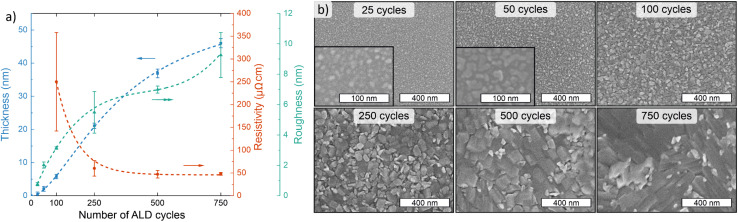
Film thickness evolution at 165 °C. (a) Thickness, resistivity, and AFM roughness, and (b) SEM images of films deposited on silicon at 165 °C *versus* the number of ALD cycles. The thickness error bars represent statistical uncertainty in the EDS measurements, while the resistivity error bars represent variation in sheet resistance over the substrate, and the roughness error bars represent standard deviation of 3–5 images per sample. All error bars correspond to one standard deviation. The lines are meant to guide the eye.

To obtain information on the film nucleation and growth, we evaluated the evolution of thickness, resistivity, roughness, and morphology as a function of film thickness at 165 °C. This deposition temperature was selected as it yields the highest growth rate, as well as the lowest resistivity and best uniformity. After a slight nucleation delay (<25 cycles), film growth was linear up to at least 250 ALD cycles, after which it seemed to slightly slow down (Fig. 3a). The decrease in the growth rate may be due to changes in morphology, namely formation of larger plate-like crystallites (Fig. 3b), or crystalline phase and orientation (Fig. S12†). The thinnest deposited films (25 and 50 cycles) were non-conductive and likely non-continuous, whereas using 100 cycles an approximately 6 nm thick conductive NiS_*x*_ film (270 μΩ cm) was deposited. With increasing thickness, the film resistivity decreased towards a stable level, reaching 64 μΩ cm at 22 nm (250 cycles) and 51 μΩ cm at 49 nm (750 cycles). The surface roughness of the NiS_*x*_ film on silicon increased rapidly from 0.16 nm for the bare substrate to 0.76 nm for the discontinuous 25 cycle film, 3.1 nm for the thinnest continuous 100 cycle film, and 9.3 nm for the 49 nm film deposited using 750 cycles (Fig. 3a; atomic force microscopy (AFM) images in Fig. S13†).

Compared to the other published NiS_*x*_ ALD processes, our NiCl_2_(TMPDA) + H_2_S process combines good ALD growth characteristics (saturation with a reasonably high growth rate and low deposition temperatures) with favorable film properties (low resistivity and impurity content), while using an affordable, easily synthesized nickel precursor (see Note S4 and Table S2† for a detailed comparison).

### High-temperature stability in different atmospheres

**Fig. 4:**
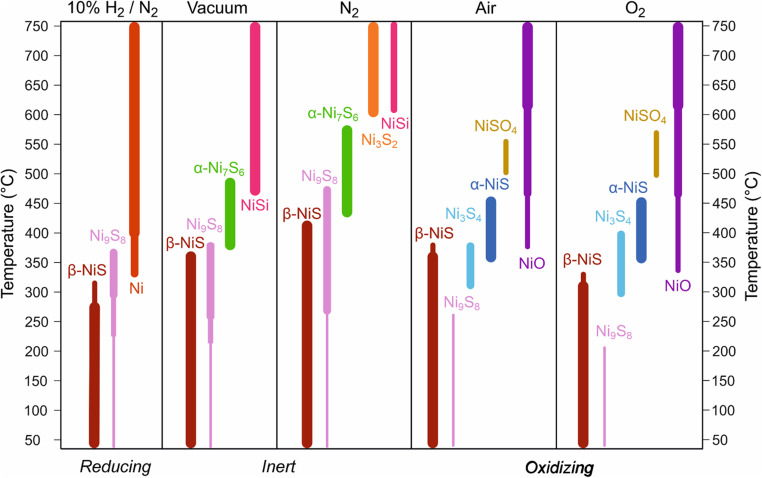
High-temperature stability. Summary of the crystalline phases observed during *in situ* grazing incidence HTXRD measurements performed in different atmospheres. The different phases are represented by the different colors. The widths of the lines represent semi-quantitative estimations on the relative amounts of the different phases based on the peak intensities.

To understand the stability of NiS_*x*_ under reducing, inert, and oxidizing conditions that are encountered in various applications and processing steps, we performed *in situ* high-temperature XRD (HTXRD) measurements in different atmospheres. Approximately 50 nm thick films deposited on Si at 165 °C that consist predominantly of β-NiS with a minor Ni_9_S_8_ phase present were used. A visual summary of the crystalline phases formed in inert, reducing, and oxidizing atmospheres is shown in Fig. 4, while the XRD data is shown in Fig. S14, S16, S18, S20 and S22.†

In a reducing forming gas environment (10% H_2_/90% N_2_, atmospheric pressure), β-NiS began to reduce at 300 °C forming Ni_9_S_8_ and gaseous H_2_S (ref. 59) (Fig. 4 and S14†). At 350 °C, metallic fcc Ni (and more H_2_S) started to form yielding a single phase of Ni metal from 425 °C until the end of the measurement at 750 °C. After cooling down from 750 °C, a discontinuous metallic Ni film was obtained (Fig. S15†). For comparison, an ALD Ni_3_N film deposited using the same nickel precursor could be reduced to metallic nickel at a temperature as low as 150 °C in 10% H_2_/N_2_ (ref. 54) and an ALD NiO film at 260 °C in 5% H_2_/N_2_.^60^ This comparison suggests that β-NiS is more difficult to reduce to Ni metal than Ni_3_N and NiO.

Under an inert N_2_ atmosphere at atmospheric pressure, the β-NiS phase was retained up to 400 °C and Ni_9_S_8_ up to 475 °C (Fig. 4 and S16†). Above this temperature, phases with a lower S/Ni ratio, first α-Ni_7_S_6_ and later Ni_3_S_2_, formed. Additionally, NiSi was observed, which is attributed to a reaction with the Si substrate (Note S5†). In a dynamic vacuum at approximately 10^−5^ mbar, similar phase transformations occurred but at ∼50–150 °C lower temperatures compared to the N_2_ atmosphere (Fig. 4, S18 and Note S6†). Although Ni_3_S_2_ was not observed during the HTXRD measurement in a vacuum, it appeared after cooling, showing that not all the sulfur was lost upon heating in a vacuum to 750 °C (Fig. S19†).

In oxidizing ambient air, Ni_9_S_8_ disappeared by 250 °C, leaving only the β-NiS phase present up to 300 °C. At 300 °C, a minor Ni_3_S_4_ component started to form (Fig. 4, S20 and Note S7†). Hexagonal α-NiS, a high-temperature form of NiS, began to form at 325 °C. Broad peaks originating from cubic NiO emerged at approximately 350 °C, although an amorphous oxide may have formed at even lower temperatures. The last sulfide phase α-NiS disappeared by 450 °C, followed by an emergence of NiSO_4_, an oxidation product of NiS.^61^ Above 550 °C only NiO was observed and its crystallinity improved with increasing temperature (Fig. S20 and S21†). When pure O_2_ was used instead of ambient air, similar phase transitions were observed at 25–50 °C lower temperatures (Fig. 4, S22 and S23†).

In summary, in a reducing atmosphere, β-NiS loses S and forms metallic Ni at 300–400 °C. This is a relatively high temperature compared to that of other nickel compounds including NiO, showing on one hand the stability of β-NiS (and other NiS_*x*_ phases) and on the other hand making reduction of β-NiS a rather inconvenient route to metallic Ni. In an inert atmosphere, S loss occurs at higher temperatures compared to the reducing conditions and remains incomplete at 750 °C. Annealing in inert atmospheres is a potential route to produce S poor phases that may be difficult to deposit directly, including Ni_9_S_8_, α-Ni_7_S_6_, and Ni_3_S_2_. Annealing in an oxidizing atmosphere yields NiO after several intermediate phases. Besides phase control by annealing, it is important to note that the temperature ranges where phase transitions occur in all of the atmospheres are similar to typical processing temperatures encountered in, for example, back end-of-line semiconductor^62^ and solar cell manufacturing (∼400 °C).^63^ Furthermore, catalysts containing NiS_*x*_ are employed for hydrotreating petroleum products, where operating temperatures of around 300–400 °C may be used.^64^

### Stability under electrochemical water splitting conditions

Besides thermal catalysis, nickel-based materials including NiS_*x*_ are considered promising catalysts for electrochemical water splitting.^65,66^ However, NiS_*x*_ is thermodynamically unstable under both acidic and alkaline water splitting conditions (see Pourbaix diagrams in Fig. S27 and S28†). The effect of electrolyte impurities is also important, especially for the OER.^34,67,68^ We evaluated both the reducing (HER) and oxidizing (OER) half reactions of water splitting in acidic (0.5 M H_2_SO_4_, pH = 0.3; *cf.* PEM electrolyzers) as well as alkaline electrolytes (0.1 M KOH, pH = 13; *cf.* alkaline and AEM electrolyzers). We deposited NiS_*x*_ thin films on fluorine-doped tin oxide (FTO) at 165 °C. The thickness of the NiS_*x*_ film was ∼15 nm for alkaline and ∼30 nm for acidic testing. The higher thickness selected in the latter case was due to thermodynamically favored dissolution in acid. The same β-NiS phase was obtained on both the Si and FTO substrates, albeit with some differences in morphology (Note S8†).

**Fig. 5:**
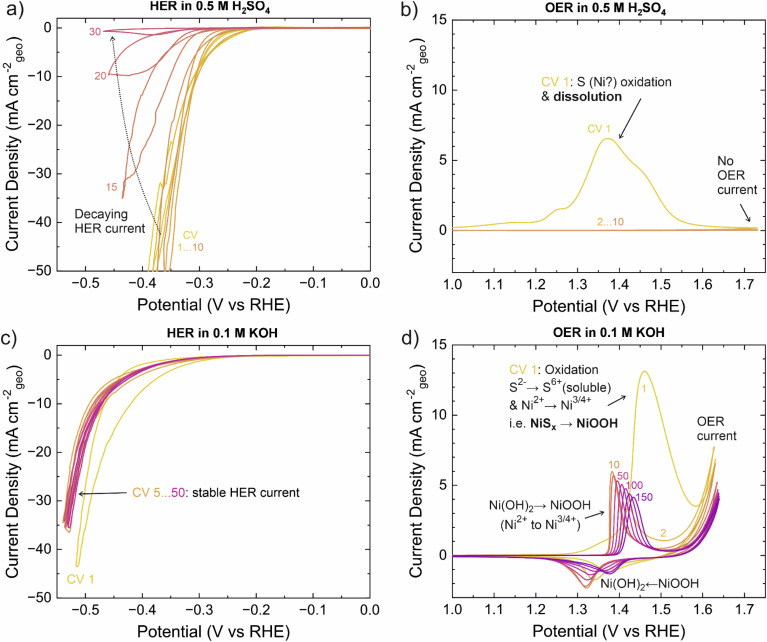
Electrochemical water splitting results. Cyclic voltammograms of NiS_*x*_ (mainly β-NiS) on FTO in different pH and voltage ranges. (a) HER and (b) OER regions in 0.5 M H_2_SO_4_ and (c) HER and (d) OER regions in 0.1 M KOH. The CVs were recorded at 10 mV s^−1^.

**Fig. 6:**
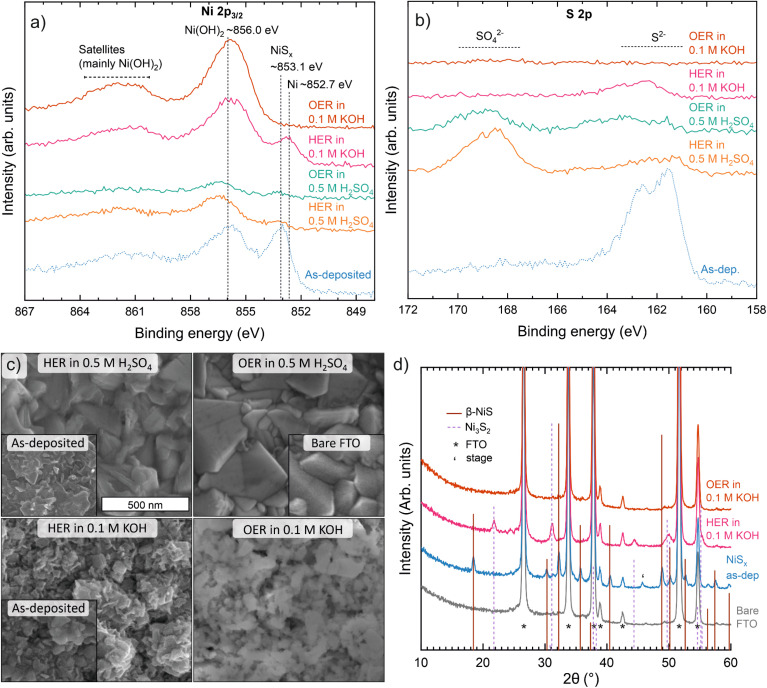
Characterization before and after electrochemistry. X-ray photoelectron spectra of (a) Ni 2p_3/2_ and (b) S 2p regions, (c) SEM images (all images at the same scale. Insets: bare FTO substrate and NiS_*x*_ films deposited using 500 ALD cycles for acidic and 250 cycles for alkaline conditions), and (d) grazing incidence XRD data of NiS_*x*_ films as deposited and after electrochemical CV experiments. Samples and electrochemical conditions for panels (a–c) are identical to Fig. 5. For (d), 400 ALD cycles were used followed by 50 CVs in the HER or OER region in 0.1 M KOH. The position and height of the lines in (d) indicate peak positions and intensities of powder references (PDF 12-41 for β-NiS and COD 9000564 for Ni_3_S_2_), while peaks from the FTO substrate and sample stage of the XRD instrument are marked with the indicated symbols.

We began the electrochemical cyclic voltammetry (CV) testing with the HER in acid (0.5 M H_2_SO_4_, between 0.0 and −0.45 V *vs.* RHE at 10 mV s^−1^). During the first CVs, relatively good catalytic activity was observed. An overpotential *η* of ∼300 mV (*i.e.* −0.3 V *vs.* RHE) was sufficient to reach the typical benchmark current density of 10 mA cm_geo_^−2^ (Fig. 5a). However, after 10 CVs (∼15 min), the HER current started to decrease and hysteresis during the CVs increased. After 30 CVs (∼45 min), the HER current was negligible, and the catalyst was removed for characterization. X-ray photoelectron spectroscopy (XPS) was used to characterize changes in the material. Although the Ni 2p_3/2_ spectra are complex, the main spectral features described in the literature allow different nickel compounds to be distinguished.^69–71^ Accordingly, the as-deposited surface was found to consist of NiS_*x*_ (Ni 2p_3/2_ binding energy (BE) ≈ 853.1 eV) and Ni(OH)_2_ (BE ≈ 856.0 eV), the latter arising from surface oxidation. After the HER experiment, Ni(OH)_2_ was observed almost exclusively (Fig. 6a). The observed S species changed from S^2−^ (S 2p_3/2_ BE ≈ 161.5 eV) to mostly SO_4_^2−^ (S 2p_3/2_ BE ≈ 168.5 eV),^72^ which is attributed to residual H_2_SO_4_ electrolyte rather than the film itself (Fig. 6b). Compared to the as-deposited sample, the intensities of both the Ni and S features were significantly weaker in relation to the Sn features from the FTO substrate (Fig. S31b†). Inductively coupled plasma optical emission spectroscopy (ICP-OES) showed that approximately two thirds of the nickel atoms were dissolved during the experiment. Furthermore, SEM showed that the surface coverage of the catalyst decreased such that the FTO substrate was exposed after the experiment (Fig. 6c). We hypothesize that the remaining NiS_*x*_ was electrically poorly connected or isolated, explaining the negligible HER current at the end of the experiment. Because a rather broad potential range was scanned during the experiments and the films were also observed to slowly dissolve under open circuit potential (OCP), a question remained whether β-NiS may still be stable under HER potentials. To this end, we applied a constant current density of 10 mA cm^−2^, which initially required an overpotential of ∼350 mV. However, *η* increased to 600 mV within 2 h (Fig. S29†), showing that the film was unstable also under constant HER operation. Thus, β-NiS appears unstable under the acidic HER conditions.

Under oxidizing conditions in acid, we observed a broad oxidation feature from 1.0 to 1.6 V *vs.* RHE and no OER current as seen in Fig. 5b. The scan was started at 0.25 V and continued up to 1.75 V *vs.* RHE; following CVs were scanned between 1.15 and 1.75 V *vs.* RHE. The oxidation feature is attributed to oxidation of sulfur (from nominally S^2−^ in β-NiS to S^6+^ in HSO_4_^−^) and its subsequent dissolution. Oxidation of Ni may also occur (*cf.* the OER under alkaline conditions), but Ni^2+^ is soluble and the thermodynamically stable species under these conditions (Fig. S27 and S28†). No significant current was observed in the following CVs, so the sample was removed for characterization after 10 CVs. ICP-OES showed that only 2% of the initial Ni remained and the sample appeared identical to the bare FTO substrate under SEM (Fig. 6c), supporting the rapid film dissolution suggested by the CVs and Pourbaix diagram. XPS showed very little Ni and S remaining that is attributed to electrically isolated domains (Fig. 6a and b). Thus, β-NiS cannot be used for the OER in acid due to its extreme instability.

Subjecting β-NiS to alkaline (0.1 M KOH) HER conditions yielded stable CVs (scanned between 0.0 and −0.55 V *vs.* RHE at 10 mV s^−1^). In the first CV, a slightly higher current and stronger hysteresis were observed (Fig. 5c), which we attribute to material changes discussed below. The CVs from the 5^th^ to 50^th^ CV (∼2 h) were practically identical. An overpotential of ∼500 mV was required to reach a current density of 10 mA cm_geo_^−2^, indicating reasonable HER activity. Post-HER characterization of the catalyst by XPS showed that the S/Ni ratio had decreased from 0.9 to 0.2 near the surface. This decrease together with a slight shift of the initial NiS_*x*_ Ni 2p_3/2_ feature (BE ≈ 853.1 eV) to 852.7 eV is attributed to formation of Ni metal and/or a sulfur-deficient Ni_3_S_2_ phase as discussed below (Fig. 6a). Furthermore, the Ni 2p_3/2_ hydroxide feature (BE ≈ 856.0 eV) increased in intensity, suggesting Ni(OH)_2_ formed either directly during the HER or when the species generated under HER conditions were exposed to air. Besides strongly decreasing in intensity, the S 2p peak shifted to a ∼0.7 eV higher BE after the HER (Fig. 6b), which may be linked to the change in the NiS_*x*_ phase or other changes in the film composition. We note that at OCP (∼0.85 V *vs.* RHE) in 0.1 M KOH, Ni(OH)_2_ (but not Ni) formation and nearly complete S loss have also been observed.^34^ The O 1s spectra showed only a single hydroxide feature that ruled out the formation of NiO (Fig. S31a†). As XPS probes surface composition, we also used EDS to determine the S/Ni ratio in the bulk of the films. The measurement showed that a significant fraction of the sulfur was lost throughout the films, resulting in a S/Ni ratio of 0.5. Grazing incidence XRD indicated that the initial β-NiS phase had completely transformed to Ni_3_S_2_ during the 50 CVs applied (Fig. 6d). As the S/Ni ratio in Ni_3_S_2_ is 0.67 compared to the observed values of 0.5 (EDS, averaging throughout the whole film) and 0.2 on the surface (XPS), the film likely also contained a non-sulfide phase, such as Ni or Ni(OH)_2_ that was too weakly crystalline to be observed by XRD. SEM also hints at the presence of two phases, as the overall plate-like morphology was retained after the HER, but small (∼10 nm) particles formed on the edges of the larger ∼100 nm wide crystallites (Fig. 6c). HER measurements of reference samples presented in Fig. S30† revealed that evaporated Ni metal was more active than β-NiS (*η* ≈ 250 mV at 10 mA cm_geo_^−2^), while Ni(OH)_2_ and NiO were less active than β-NiS (*η* ≈ 510 and >600 mV at 10 mA cm_geo_^−2^). The Pourbaix diagram suggests Ni to be the stable species under alkaline HER conditions (Fig. S27 and S28†). Resolving the HER active species likely requires a multi-technique *in situ*/*operando* structural and compositional investigation.^41^ Regardless, our results highlight the instability of β-NiS and its bulk transformation to Ni_3_S_2_ and potentially additional surface Ni metal or Ni(OH)_2_ species under alkaline conditions.

Finally, we explored alkaline OER conditions (0.1 M KOH, CVs in 0.85 to 1.65 V *vs.* RHE range at 10 mV s^−1^). During the forward (anodic) scan of the first CV, a large irreversible oxidation feature was observed at ∼1.2–1.6 V *vs.* RHE (Fig. 5d). We attribute this feature to oxidation of both nickel (from Ni^2+^ in β-NiS to a mixture of Ni^3+^ and Ni^4+^ in “NiOOH”) and sulfur (from S^2−^ in β-NiS to S^6+^ in SO_4_^2−^). The integrated peak area determined after deduction of the OER current was within 10% of the theoretically expected amount of transferred charge for the proposed oxidation reaction. The generated sulfate is soluble, while the NiOOH is not and functions as an OER catalyst.^67,68,73^ On the reverse (cathodic) scan, the reduction feature observed at ∼1.35 V is attributed to reversible NiOOH → Ni(OH)_2_ transformation. During repeated CVs, the reversible Ni(OH)_2_/NiOOH redox features remain, although they shift to higher potentials and decrease in area from CV 10 to CV 150, implying changes in the structure of NiOOH and likely incorporation of iron impurities (see below).^67,73,74^ Regarding the Ni(OH)_2_/NiOOH phase, the position (1.38 V *vs.* RHE for the oxidation peak) and area (1.7 e^−^ per Ni atom for the reduction peak) at CV 10 are in agreement with the α-Ni(OH)_2_/γ-NiOOH redox couple.^34,68,73,74^ The decrease in area to 1.0 e^−^ per Ni atom by CV 150 corresponds to formation of the β-Ni(OH)_2_/β-NiOOH couple, which is spontaneous in an alkaline electrolyte,^68,73^ possibly accompanied by trace Fe incorporation (see below). A slight decrease in the OER current was observed during the experiment, but the current stabilized to approximately 3 mA cm^−2^ at 1.63 V *vs.* RHE (*i.e. η* = 400 mV). XPS showed that only Ni(OH)_2_ was present after the OER experiment (Ni 2p_3/2_ BE ≈ 856.0 eV, Fig. 6a; O 1s BE = 531.3 eV, Fig. S31a†), which is in line with ending the measurement below the NiOOH/Ni(OH)_2_ reduction potential as well as the instability of NiOOH in air.^75^ No sulfur remained in the films according to XPS (Fig. 6b) and EDS that probe the surface and the whole film, respectively. In contrast, no signs of dissolution of Ni were observed, in accordance with the previous ICP-OES studies.^34^ No reflections from the film were detected by XRD (only those from the substrate) after the OER, which suggests that the formed (oxy)hydroxide has low crystallinity and is in line with the complete disappearance of NiS_*x*_. In terms of the morphology, SEM suggested the formation of a more porous structure that retained the general plate shape of the initial NiS_*x*_ crystallites (Fig. 6c). We note that iron impurities are known to easily incorporate into nickel-based OER catalysts and have a beneficial effect on their activity.^67,68,76^ Although the concentration of Fe was below our XPS detection limit of ∼2 cation-% (based on scans in the Fe 3p region), we believe Fe impurities to play a role in supporting a relatively stable OER current as our experiments were performed in reagent grade KOH.^67,68^ The observed shift of the Ni redox features to higher potentials and the decrease in area during consequent CVs being stronger compared to Ni(OH)_2_ and NiS_*x*_ measured in purified KOH in the literature also support Fe incorporation (Fig. 5d).^34,67,68,77^ Thus, under the alkaline OER conditions β-NiS transforms to Ni(Fe)OOH, a well-known OER catalyst. Its OER activity can be significantly improved by introducing an elevated level of Fe impurities into the alkaline electrolyte.^34^

In summary, both the electrochemical potential and electrolyte pH play crucial roles in defining the stability of the NiS_*x*_ electrocatalyst. Importantly, structural changes occurred under all of the studied conditions (HER, OER, and OCP in acid and base). Our β-NiS films were found to be unstable under acidic conditions, ranging from slow dissolution under reductive potentials (HER) and OCP to very fast dissolution under oxidizing conditions (OER). Thus, the results suggest that β-NiS is a poor choice for acidic water splitting, *i.e.* PEM electrolyzers. Our results are in line with the solubility of Ni^2+^ in acid. Under alkaline conditions, dissolution of Ni was not observed but sulfur loss occurred. Under reducing conditions, β-NiS “bulk” transformed to Ni_3_S_2_ with nickel metal and/or Ni(OH)_2_ potentially forming at least on the surface, while under oxidizing conditions the whole film transformed to (oxy)hydroxide. The oxyhydroxide formation from NiS_*x*_ under OER conditions has been observed in several studies.^22,25,32,33^ While at least partial sulfur loss under HER conditions has been found in multiple studies,^35–40^ no consensus has been reached on what species are formed with suggestions including Ni_3_S_2_,^35,36^ Ni metal,^37^ NiO_*x*_S_*y*_,^38^ and Ni(OH)_2_ (ref. 39 and 40). The different species observed may stem from differences in the precatalyst properties (composition, structure, morphology *etc.*), testing conditions (different potentials in cyclic voltammetry, chronoamperometry, and chronopotentiometry), and characterization methods (both *ex situ* and *in situ*, each with different capabilities to observe different materials). We observed the formation of Ni_3_S_2_ in agreement with ref. 35 and 36. From the post-HER characterization we infer that a fraction of Ni and/or Ni(OH)_2_ may also form as suggested in ref. 37, 39 and 40. Factors such as the catalyst phase and morphology as well as the substrate affect the electrochemical stability; however, both the calculated^27,78^ and experimental thermodynamic data suggest that all of the NiS_*x*_ phases are slightly and strongly unstable under the HER and OER conditions, respectively (Fig. S27 and S28†). The observed Ni_3_S_2_ is the most stable (or least unstable) sulfide under reducing conditions, but Ni metal is thermodynamically the most stable species under alkaline HER conditions. Under OER conditions, NiOOH is predicted to be stable, in line with our observations.

We can now compare the stability of NiS_*x*_ under electrochemical water splitting conditions to annealing in oxidizing and reducing atmospheres (see the section High-temperature stability in different atmospheres). Under both kinds of reducing conditions, *i.e.* HER and annealing in H_2_, we observed the reduction of Ni and decrease of S content, which during H_2_ annealing produced Ni metal. During the alkaline HER, mostly Ni_3_S_2_ formed with hints of Ni metal and Ni(OH)_2_ formation. Under oxidizing conditions, *i.e.* OER and annealing in air or O_2_, NiS_*x*_ oxidized to NiO_*x*_(H_*y*_). The electrochemical oxidation in the presence of water results in the (oxy)hydroxide instead of the thermally formed oxide. Furthermore, the OER conditions present a stronger driving force, forming Ni^3+/4+^ instead of Ni^2+^ during annealing in air or O_2_. Solubility and evaporation are additional key factors that are present in aqueous environments and annealing only, respectively. Thermal annealing has dramatic effects on morphology due to fast diffusion processes, while this is usually absent in aqueous solutions. Finally, we note that our approach of using thin film catalysts coupled with pre/post characterization is broadly applicable to different materials and applications.

## Conclusions

We have developed a new nickel sulfide ALD process using easily synthesized NiCl_2_(TMPDA) and H_2_S at 165–225 °C. The process compares favorably with previously reported processes, depositing uniform and conformal, highly conductive films with low impurity levels that consist mainly of the β-NiS phase with a minor Ni_9_S_8_ component. The effects of the deposition temperature and film thickness on film properties were characterized. The highest growth rate, lowest resistivity, and highest amount of the β-NiS phase were observed at 165 °C. Continuous, conductive films were obtained from a thickness of 6 nm at 165 °C, with an increase in thickness decreasing resistivity and increasing roughness. With increasing deposition temperature, the amount of the Ni_9_S_8_ phase increased. High-temperature XRD measurements in reducing, inert, and oxidizing atmospheres revealed that the films undergo structural and compositional changes starting from 300–400 °C, *i.e.* temperatures relevant to processing and operation of various applications. The material stability was systematically assessed under electrochemical water splitting conditions. In acid, β-NiS was found to dissolve under the HER and OER, as well as OCP conditions. Under alkaline conditions, rather stable HER and OER currents were obtained, but material characterization and electrochemical data suggested the sulfide to transform to Ni(OH)_2_ at OCP and NiOOH during the OER. During the HER, Ni_3_S_2_ formed, possibly with additional Ni and/or Ni(OH)_2_ species. These observations highlight the importance of characterizing the stability of sulfides and other materials under application-relevant processing and operating conditions. Beyond fundamental stability studies, our ALD process provides a scalable method for engineering advanced NiS_*x*_ nanostructures on high surface area substrates for various thermal and electrochemical catalytic processes and beyond.

## Experimental

### Film deposition

Nickel sulfide films were deposited using a commercial, hot-wall, cross-flow ALD reactor (F120, ASM Microchemistry) operated at approximately 5 mbar pressure.^79^ Nitrogen (N_2_, 99.999%, AGA) was further purified using a SAES Microtorr MC1-902F purifier and used as the carrier and purge gas at a flow rate of 400 sccm. The dichloro(*N*,*N*,*N*′,*N*′-tetramethyl-1,3-propanediamine)nickel(ii) [NiCl_2_(TMPDA)] precursor synthesized in house^53^ was heated to 157 °C in an open boat held inside the ALD reactor and pulsed by inert gas valving. The vapor pressure of NiCl_2_(TMPDA) was estimated to be 0.1 mbar at 157 °C.^55^ The moderately air sensitive precursor was stored and handled in a N_2_ glovebox before transferring to the ALD reactor. The reactant hydrogen sulfide (H_2_S, 99.5%, Linde) was further purified using a SAES Microtorr MC1-302F purifier. The flow rate of H_2_S was set to 14 sccm under a continuous flow using a mass flow meter and a needle valve and it was pulsed into the reactor using an external solenoid valve.

**Caution!** Safe use of highly toxic and flammable H_2_S gas requires a properly designed ALD reactor and laboratory space. The H_2_S bottle was stored in a ventilated gas cabinet and the H_2_S lines were designed to be compatible with H_2_S, using VCR metal and EPDM polymer seals. The reactor exhaust was bubbled through an aqueous Cu(NO_3_)_2_ solution to remove the H_2_S downstream of the vacuum pump by precipitation. Reactor modifications required for H_2_S compatibility have been discussed by Dasgupta *et al.*^80^

The NiS_*x*_ films were mostly deposited on 5 × 5 cm^2^ Si(100) and soda lime glass (SLG) substrates. FTO coated glass (15 × 20 mm^2^, TEC 8, Ossila) was used to prepare samples for electrocatalysis. The silicon substrates with a native oxide layer were used as supplied. The SLG substrates were cleaned using successive ultrasonic baths of alkaline detergent (Industrial Strength Cleaner, Branson), tap water, deionized water, and ethanol (10 minutes each, room temperature) followed by careful rinsing using deionized water and a 50 : 50 (v/v%) deionized water/ethanol solution and blown dry using pressurized air. The FTO substrates were cleaned analogously, except that isopropanol was used in the last ultrasonication and rinsing steps.

### Film characterization

The film morphology was examined by SEM (Hitachi S-4800 and FEI Magellan 400 XHR) and AFM (Veeco Multimode V). Tapping mode AFM imaging was performed in air using silicon probes with a nominal radius of less than 10 nm (Bruker). The AFM images were flattened with no other image processing and film roughness was calculated as a root-mean-square (*R*_q_) value.

Film thicknesses were measured by EDS (Oxford INCA 350 connected to the Hitachi S-4800 SEM). GMRFilm software^81^ was used to convert the measured Ni and S Kα *k*-ratios to film thicknesses assuming bulk density of β-NiS (5.5 g cm^−3^).^82^ Attempts to use X-ray reflectivity to confirm the thickness or density of the films were unsuccessful due to their high roughness. Sheet resistance was measured using a four-point-probe (CPS Probe station connected to a Keithley 2400 SourceMeter). The sheet resistance was converted to resistivity using the thickness measured by EDS.

Crystallinity was studied by XRD (Rigaku SmartLab) using Cu Kα radiation (*λ* = 1.54 Å) in both grazing incidence (*ω* = 1°) and *θ*–2*θ* geometries. Raman spectroscopy was also used to evaluate phase composition. An NT-MDT Ntegra instrument was used in the back-scattering geometry using a 100× objective and 532 nm laser with a nominal power of 20 mW.

Film composition was analyzed by ToF-ERDA using a 40 MeV ^127^I^7+^ ion beam. The incident beam-sample and sample-recoiled beam angles were 16 and 24°. The film surface and film/substrate interfaces were excluded from the analysis. The surface composition and chemical state and changes in them after the electrochemical measurements were analyzed by XPS (PHI VersaProbe 3) using monochromatized Al Kα radiation (*hν* = 1486.6 eV). The photoelectron take-off angle and incoming X-ray angle were 45° and 90° with respect to the sample surface. No sputtering was performed. A charge neutralizer was used during the measurements. The spectra were referenced to the C 1s C–C component of adventitious carbon at 284.8 eV. Pass energy was set to 55 and 224 eV for the core and survey scans. The S/Ni ratios were calculated using PHI Multipak software by integrating the measured Ni 2p_3/2_ and S 2p spectra using Shirley backgrounds and relative sensitivity factors provided with the software.

Selected samples were digested before and after the electrochemical measurements followed by analysis of the nickel concentration using ICP-OES (Thermo Scientific ICAP 6300 Duo View). The digestion was done in a Teflon compression cell where the analysis area was limited using an O-ring (9 mm internal diameter), adding 400 μL of concentrated HNO_3_ (TraceMetal grade, Fisher Chemical), which was let to react for at least 2 h followed by collection and dilution to 5% HNO_3_ using ultrapure H_2_O. Standards were prepared from 1000 mg per L stock solutions to the desired concentrations (∼10–1000 μg L^−1^) in 5% HNO_3_.

### High-temperature XRD

The stability of the NiS_*x*_ films under different atmospheres was studied by *in situ* HTXRD measurements using an Anton-Paar HTK1200N oven connected to a PANalytical X'Pert Pro MPD diffractometer using Cu Kα radiation (*λ* = 1.54 Å) in the grazing incidence (*ω* = 1°) geometry. Different atmospheres were used, including atmospheric pressure of laboratory air, O_2_ (AGA, 99.999%), N_2_ (AGA, 99.999%, further purified using an Entegris Gatekeeper purifier), and forming gas (10% H_2_ in N_2_, AGA), as well as dynamic vacuum (approximately 10^−5^ mbar). The studied NiS_*x*_ films (deposited at 165 °C using 750 cycles) were first heated from room temperature up to 125 °C at a heating rate of 10 °C min^−1^. Thereafter, the heating was paused every 25 °C to record a diffractogram, which took 27 min. The measurements were finished at 750 °C, resulting in a total measurement time of approximately 15 h.

### Electrochemical measurements

Electrochemical measurements were performed using a three-electrode poly(tetrafluoroethylene) (PTFE) compression cell^83,84^ and a BioLogic SP-200 potentiostat. ALD NiS_*x*_ deposited on FTO-coated glass was used as a working electrode and placed underneath the cell such that an O-ring exposed an area of 0.5 cm^2^ to the electrolyte. Electrical connection was made using Cu tape to a section of the working electrode not exposed to the electrolyte. A coiled Pt wire was stored in 60% HNO_3_, rinsed with ultrapure water (Milli-Q, resistivity 18.2 MΩ cm), flame-annealed, and then used as a counter electrode. A glass-body Ag/AgCl reference electrode (4 M KCl with saturated Ag, Fisherbrand Accumet 13-620-53) was used. The potential of the Ag/AgCl reference electrode was referenced to a commercial reversible hydrogen electrode (RHE, Gaskatel HydroFlex).

Measurements were performed under both alkaline and acidic conditions. For the alkaline measurements, a 0.1 M KOH electrolyte (pH = 13) was prepared from granular KOH (ACS Reagent, Sigma-Aldrich, <0.001% Fe, which corresponds to <70 ppb after dilution to 0.1 M) and ultrapure water with the help of a pH meter and a 0.1 M KOH standard (Titripur, Supelco). No additional electrolyte purification was performed. Although no Fe was observed on the samples by XPS after the electrochemical measurements, we expect some iron to incorporate into the samples.^67,74^ For the acidic measurements, a 0.5 M H_2_SO_4_ electrolyte (pH = 0.3) was prepared from concentrated H_2_SO_4_ (TraceMetal grade, Fisher) and ultrapure water.

Prior to starting the electrochemical measurements, approximately 20 mL of the electrolyte was added to the cell described above, the electrodes were installed, and the cell was closed with a lid and sparged with N_2_ using a glass gas dispersion tube for 15 min before starting the measurement. The sparging was continued throughout the experiment, while no additional stirring was performed.

Typical electrochemical measurements consisted of cyclic voltammetry (CV) scans at 10 mV s^−1^. The potential ranges were 0 to −0.7 V *vs.* Ag/AgCl (HER in 0.5 M H_2_SO_4_), −0.9 to −1.6 V *vs.* Ag/AgCl (HER in 0.1 M KOH), 0.9 to 1.5 V (OER in 0.5 M H_2_SO_4_), and −0.1 to 0.7 V *vs.* Ag/AgCl (OER in 0.1 M KOH). Depending on the film stability, 10 (OER in 0.5 M H_2_SO_4_) to 150 CVs (OER in 0.1 M KOH) were applied. The CVs compensated for 85% of the *iR*_u_ drop during the measurements, while the remaining 15% was compensated for during post-processing. All the shown data are therefore 100% *iR*_u_ drop compensated. *R*_u_ was measured every 3 CVs using EIS at −0.1 V *vs.* Ag/AgCl, 5 kHz frequency (chosen for phase angle close to 0), and 10 mV root-mean-square amplitude. The measured *R*_u_ was ∼40 Ω in 0.1 M KOH and ∼10 Ω in 0.5 M H_2_SO_4_. After the measurements, the samples were rinsed with ultrapure H_2_O and stored in air for characterization.

## Conflicts of interest

There are no conflicts of interest to declare.

## Supplementary Material

TA-013-D5TA00663E-s001

## Data Availability

The data supporting this article have been included as part of the ESI.† Additional data may be requested from the authors.
