# Supplementary material for: Atomic layer deposition of nickel sulfide thin films and their thermal and electrochemical stability
Source: J Mater Chem A Mater. 2025 Jul 10;13(31):25703–14. doi: 10.1039/d5ta00663e (PMC12243761; doi:10.1039/d5ta00663e)
Supplement: TA-013-D5TA00663E-s001 [file TA-013-D5TA00663E-s001.pdf]

Supplementary Information (SI) for Journal of Materials Chemistry A.
This journal is © The Royal Society of Chemistry 2025

Supplementary Information for:

Atomic Layer Deposition of Nickel Sulfide Thin Films and Their Thermal and Electrochemical Stability

Miika Mattinen,*[†‡] Johanna Schröder,[‡⁋Δ] Timo Hatanpää,[†] Georgi Popov,[†] Kenichiro Mizohata,[§] Markku Leskelä,[†] Thomas F. Jaramillo,[‡°⁋] Michaela Burke Stevens,[⁋] Stacey F. Bent,[‡°] and Mikko Ritala*[†]

† Department of Chemistry, University of Helsinki, P.O. Box 55, FI-00014, Finland

‡ Department of Chemical Engineering, Stanford University, 443 Via Ortega, Stanford, California 94305, United States of America

⁋ SUNCAT Center for Interface Science and Catalysis, SLAC National Accelerator Laboratory, 2575 Sand Hill Road, Menlo Park, California 94025, United States of America

§ Division of Materials Physics, Department of Physics, University of Helsinki, P.O. Box 43, FI-00014, Finland

° Department of Energy Science and Engineering, Stanford University, 443 Via Ortega, Stanford, California 94305, United States of America

Δ Present address: Institute for Chemical Technology and Polymer Chemistry (ITCP), Karlsruhe Institute of Technology (KIT), Engesserstraße 18, 76131 Karlsruhe, Germany

*email: miika.mattinen@helsinki.fi (M.M.) mikko.ritala@helsinki.fi (M.R.)

Contents

Section S1. Additional experiments on ALD characteristics **S3**
Figure S1. SEM images and thicknesses of $NiCl_2$(TMPDA) decomposition experiments S3
Figure S2. S/Ni atomic ratio versus pulse lengths and deposition temperature S3
Figure S3. Growth rate, resistivity, and S/Ni atomic ratio versus purge length S3
Figure S4. SEM image demonstrating conformal coverage of NiS_x on a trench structure S4

Section S2. Effect of deposition temperature on film characteristics **S5**
Note S1. Raman analysis S5
Figure S5. Raman spectra of NiS_x films deposited at different temperatures S5
Table S1. Elemental composition of NiS_x films deposited at different temperatures (ToF-ERDA) S5
Figure S6. ToF-ERDA elemental depth profiles of films deposited at different temperatures S6
Note S2. Preferred orientation of NiS_x S6
Figure S7. θ-2θ X-ray diffractograms of films deposited at different temperatures S7

Section S3. Effect of pulse times on film characteristics **S8**
Note S3. Effect of pulse times on film characteristics S8
Figure S8. SEM images of films deposited with different $NiCl_2$(tmpda) pulse lengths S8
Figure S9. SEM images of films deposited with different H_2S pulse lengths S8
Figure S10. X-ray diffractograms of films deposited with different $NiCl_2$(tmpda) pulse lengths S9
Figure S11. X-ray diffractograms of films deposited with different H_2S pulse lengths S9

Section S4. Effect of thickness on film characteristics **S10**
Figure S12. X-ray diffractograms of films deposited with a different number of ALD cycles at 165 °C S10
Figure S13. AFM images of films deposited at 165 °C with a different number of ALD cycles S10

Section S5. Comparison to other NiS_x ALD processes **S11**
Note S4. Comparison to other NiS_x ALD processes. S11
Table S2. Reported NiS_x ALD processed and selected film characteristics S11

Section S6. HTXRD measurements and post-characterization **S12**
Figure S14. High-temperature diffractograms in a forming gas atmosphere from 25 to 750 °C S12
Figure S15. X-ray diffractogram and SEM images after heating in forming gas up to 750 °C S12
Note S5. Annealing in N_2 (inert) atmosphere S12
Figure S16. High-temperature diffractograms in a N_2 atmosphere from 25 to 750 °C S13
Figure S17. X-ray diffractogram and SEM images after heating in N_2 up to 750 °C S13
Note S6. Annealing in vacuum (inert) atmosphere S13
Figure S18. High-temperature diffractograms in a vacuum from 25 to 750 °C S14
Figure S19. X-ray diffractogram and SEM images after heating in a vacuum up to 750 °C S14
Note S7. Annealing in air and O_2 (oxidizing) atmospheres S14
Figure S20. High-temperature diffractograms in air from 25 to 750 °C S15
Figure S21. X-ray diffractogram and SEM images after heating in air up to 750 °C S15
Figure S22. High-temperature diffractograms in an O_2 atmosphere from 25 to 750 °C S16
Figure S23. X-ray diffractogram and SEM images after heating in O_2 up to 750 °C S16

Section S7. Characterization of films deposited on FTO vs Si **S17**
Note S8. Comparison of NiS_x growth and properties on FTO and Si S17
Figure S24. Thickness on Si and FTO substrates by EDS S17
Figure S25. Crystallinity on Si and FTO substrates by XRD and Raman S17
Figure S26. Morphology on Si and FTO substrates by SEM S18

Section S8. Pourbaix diagrams **S19**
Note S9. Pourbaix diagrams S19
Figure S27. Pourbaix diagram for aqueous NiS system using data calculated in the Materials Project S19
Figure S28. Pourbaix diagrams for aqueous Ni-S system based on experimental data (HSC Chemistry) S20

Section S9. Electrochemical experiments and before/after characterization **S21**
Figure S29. Chronopotentiometry of a NiS_x film on FTO at 10 mA/cm^2 in 0.5 M H_2SO_4 S21
Figure S30. Comparison of different Ni-based materials for HER in 0.1 M KOH S21
Figure S31. XPS of NiS_x films as deposited and after electrochemical CV experiments S22

Section S10. References **S23**

Section S1. Additional experiments on ALD characteristics

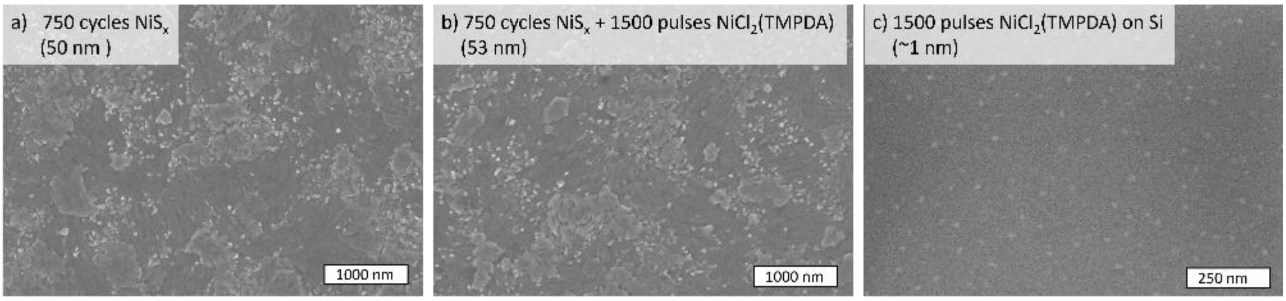

Figure S1. SEM images and EDS thicknesses of a) as-deposited NiS_x film (165 °C, 750 cycles, 2.0 s $NiCl_2$(tmpda) and H_2S pulses with 1.0 s purges), b) the NiS_x film after 1500 additional four-second $NiCl_2$(tmpda) pulses, and c) bare silicon substrate after 1500 four-second $NiCl_2$(tmpda) pulses. All pulses were applied at 165 °C. The 3 nm thickness increase from a) to b) and the ~1 nm thickness of film deposited in c) shows the decomposition of $NiCl_2$(TMPDA) at 165 °C is negligible.

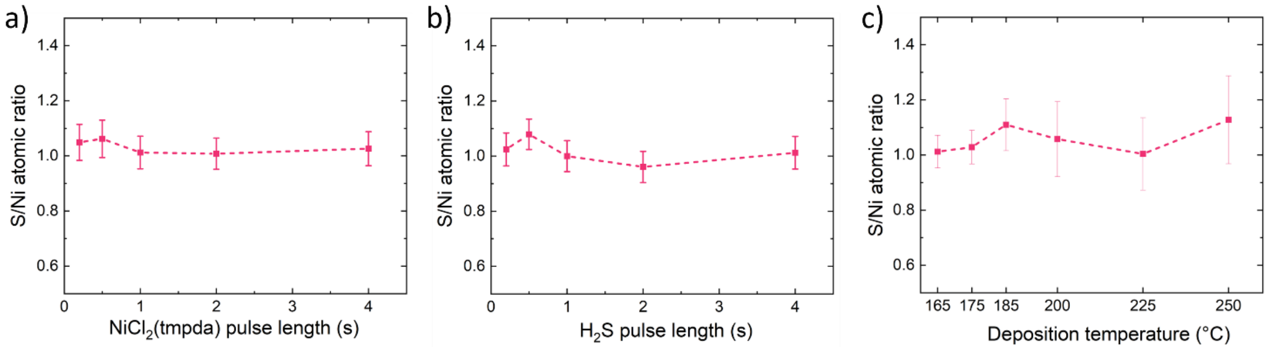

Figure S2. S/Ni atomic ratio determined by EDS versus a) $NiCl_2$(tmpda) and b) H_2S pulse length and c) deposition temperature. The error bars refer to statistical measurement uncertainty (one standard deviation). The films were deposited on silicon using, unless otherwise noted, 750 cycles with 2.0 s $NiCl_2$(tmeda) and 4.0 s H_2S pulses separated by 2.0 s N_2 purges at 165 °C.

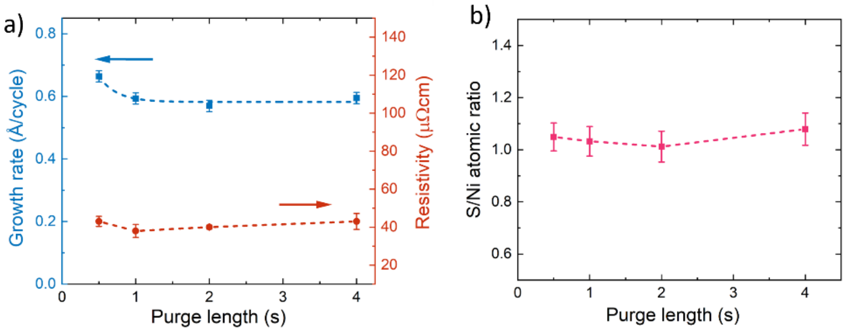

Figure S3. a) Growth rate and resistivity and b) S/Ni atomic ratio versus purge length. The error bars in growth rates and S/Ni atomic ratios represent statistical measurement uncertainty (one standard deviation) in EDS measurement. The resistivity error bars represent variation (standard deviation) in square resistance over the substrate (i.e. calculated using a single thickness value per sample). The films were deposited on silicon using 750 cycles with 2.0 s $NiCl_2$(tmeda) and 4.0 s H_2S pulses at 165 °C.

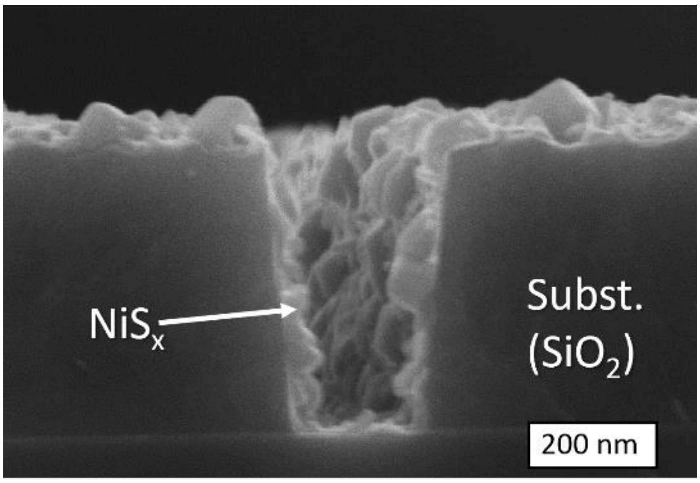

Figure S4. SEM image demonstrating conformal coverage of NiS_x on a trench structure with an aspect ratio of approximately two. The trench is slightly tilted from right to left in the image and a part of the rough film was removed when the sample was prepared by cleaving. The film was deposited at 165 °C using 375 cycles with 4.0 s $NiCl_2$(tmpda) and H_2S pulses separated by 5.0 s purges.

Section S2. Effect of deposition temperature on film characteristics

Note S1. Raman analysis.

Raman spectroscopy showed modes attributed to β-NiS (Millerite) at all deposition temperatures (Figure S5).[1] The weak 188 cm^{-1} mode observed in the 225 °C sample may perhaps be attributed to Ni_3S_2.[2] The strongest reported modes of Ni_3S_2 overlap with those of β-NiS, making verification of the presence of Ni_3S_2 challenging. However, XRD did not indicate formation of Ni_3S_2 (Figure 2). No reliable reference data could be found for Ni_9S_8 or Ni_7S_6 which were observed by XRD (for at least some of the samples), suggesting that these phases are weakly or not at all Raman active.[2,3] Comparison to available reference data showed that no Raman modes attributable to NiO (Raman Open Database, entry 3500095),[4] NiS_2,[5] or Ni_3S_4(Ref.[6]) were observed.

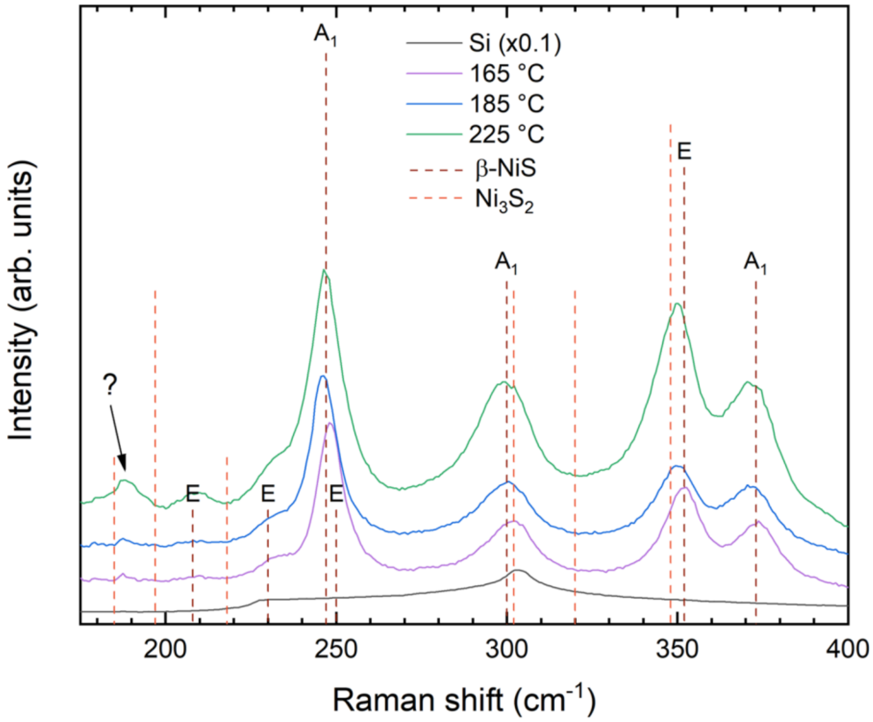

Figure S5. Raman spectra of 44–50 nm thick films deposited on Si at different temperatures (165 °C: 750 cycles, 185 °C: 1500 cycles, 225 °C: 2250 cycles; 2.0 s $NiCl_2$(TMPDA) and H_2S pulses and purges) and bare Si substrate. Literature positions for β-NiS (Ref.[1]) and Ni_3S_2 (Ref.[2]) are shown with the height of the dashed lines corresponding to reported relative intensities.

Table S1. Elemental composition of NiS_x films deposited on silicon at different temperatures analyzed by ToF-ERDA. Interfaces with substrate and air were excluded from analysis. The films were deposited using 750 (165 °C), 1500 (185 °C), or 2250 (225 °C) cycles consisting of 2.0 s $NiCl_2$(tmeda) and H_2S pulses and purges.

Deposition temperature (°C)	Ni (at-%)	S (at-%)	S/Ni	Cl (at-%)	O (at-%)	C (at-%)	N (at-%)	H (at-%)
165	48.2±0.7	49.4±0.8	1.02±0.01	<0.93	0.23±0.02	0.31±0.05	0.08±0.03	0.84±0.11
185	48.4±0.7	49.4±0.8	1.02±0.01	<0.83	0.30±0.03	0.27±0.03	0.02±0.01	1.23±0.10
225	48.8±0.7	48.7±0.8	1.00±0.01	<0.92	0.36±0.04	0.52±0.08	0.07±0.06	0.60±0.08

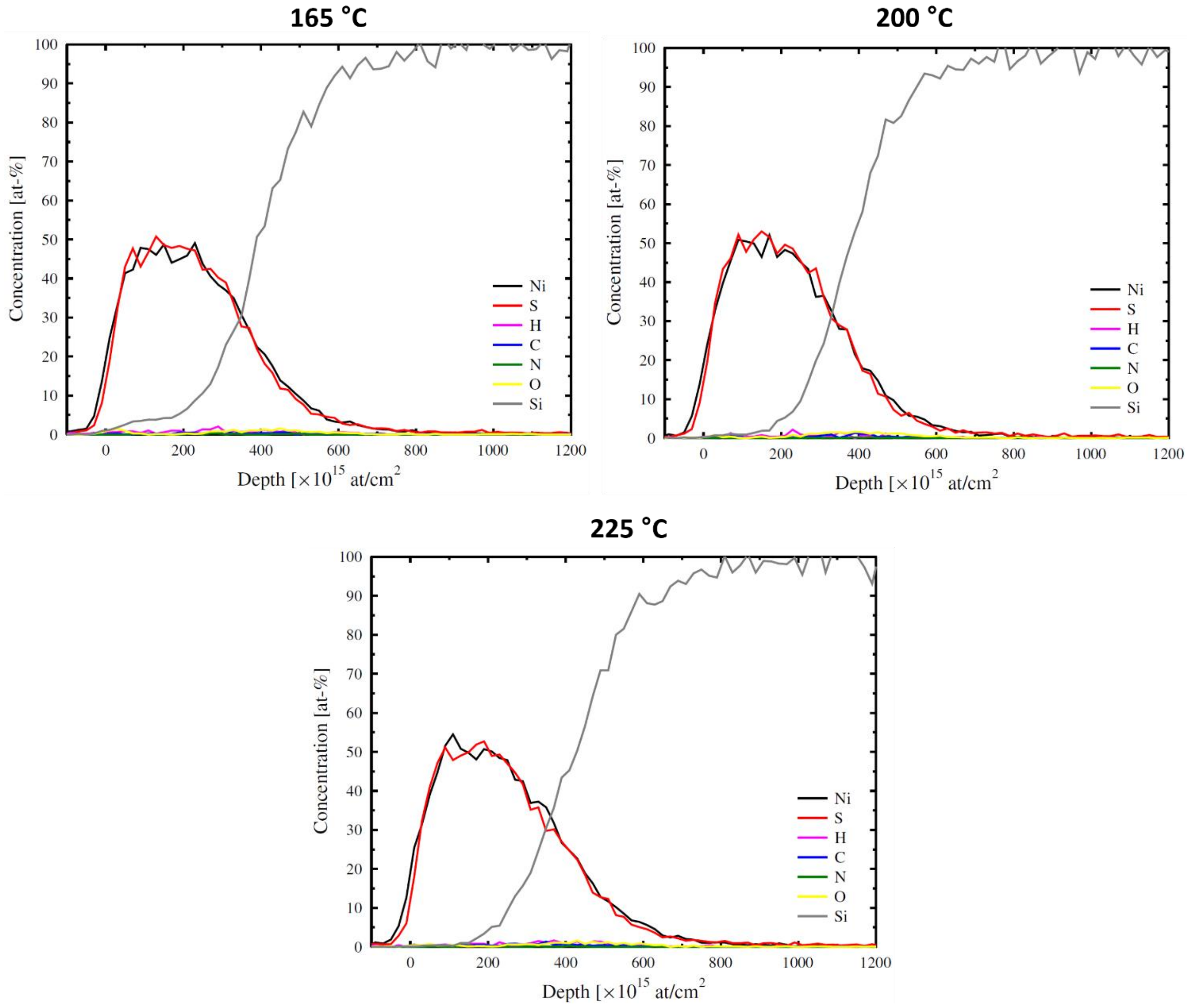

Figure S6. ToF-ERDA elemental depth profiles of films deposited at different temperatures. The films were deposited on silicon using 750 (165 °C), 1500 (200 °C), or 2250 cycles (225 °C) consisting of 2.0 s $NiCl_2$(tmeda) and H_2S pulses and purges.

Note S2. Preferred orientation of NiS_x.

In θ-2θ XRD measurements that probe planes parallel to substrate surface, the intensities of the β-NiS phase differ from the reference intensities for randomly oriented material, which are 60% (110), 40% (101), 100% (300), 45% (410) relative to the (300) reflection (PDF 12-41). In addition, several strong reflections including (131) at 48.8 °2θ are completely missing in our films (see Figure S7 showing reference intensities). Thus, the β-NiS phase in the films shows preferred (300) orientation. Similarly, the Ni_9S_8 phase has a preferred (111) orientation particularly at 225 °C and above. This is apparent from there reference data (PDF 22-1193), where the relative intensity of the (243) reflection is 30% relative to the (222) reflection, while other strong reflections are missing from our films, including (202) at 27.2 °2θ (50%), (241) at 38.6 °2θ (40%), (024) at 41.4 °2θ (40%), (043) at 43.0 °2θ (50%), and (530) at 55.5 °2θ (80%).

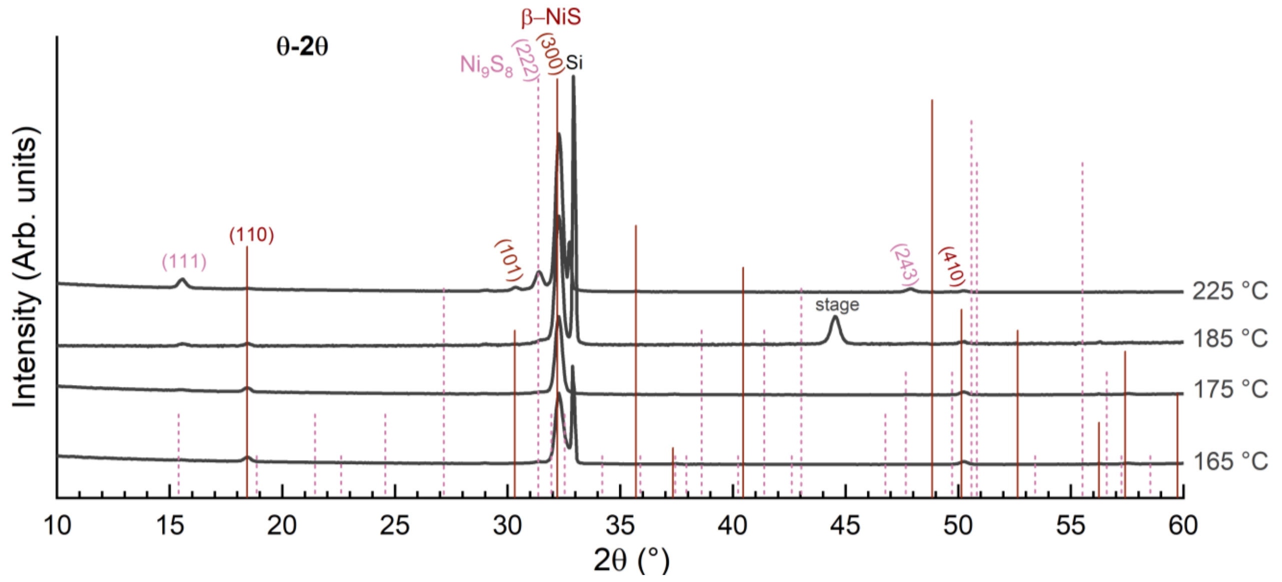

Figure S7. θ-2θ X-ray diffractograms of 46-50 nm thick films deposited at different temperatures. The films were deposited using 750 (165, 175 °C), 1500 (185 °C), and 2250 cycles (225 °C) and 2.0 s $NiCl_2$(tmeda) and H_2S pulses and N_2 purges. The position and height of the lines indicate peak positions and intensities of powder references (PDF 12-41 for β-NiS and 22-1193 for Ni_9S_8). Peaks originating from the Si substrate and sample stage of the XRD instrument are also indicated.

Section S3. Effect of pulse times on film characteristics

Note S3. Effect of pulse times on film characteristics.

When increasing the $NiCl_2$(TMPDA) pulse length at 165 °C, slight changes in the morphology (Figure S8) and an increase in the intensity of Ni_9S_8 peaks were observed (Figure S10). The changes at $NiCl_2$(TMPDA) pulse lengths of 1.0 s and above were minor. Regardless, these changes may explain the somewhat atypical saturation behavior (Figure 1a). Increasing the H_2S pulse length from 0.2 to 2.0 s had a qualitatively opposite effect on morphology compared to the $NiCl_2$(TMPDA) pulse length (Figure S9). The amount of Ni_9S_8 slightly increased with increasing H_2S pulse length. The changes in morphology and phase composition are likely linked to the observed decrease in growth rate and resistivity upon increasing the H_2S pulse length (Figure 1b). However, most of the changes occurred in the sub-saturation regime (<1.0 s pulses), as expected for a good ALD process.

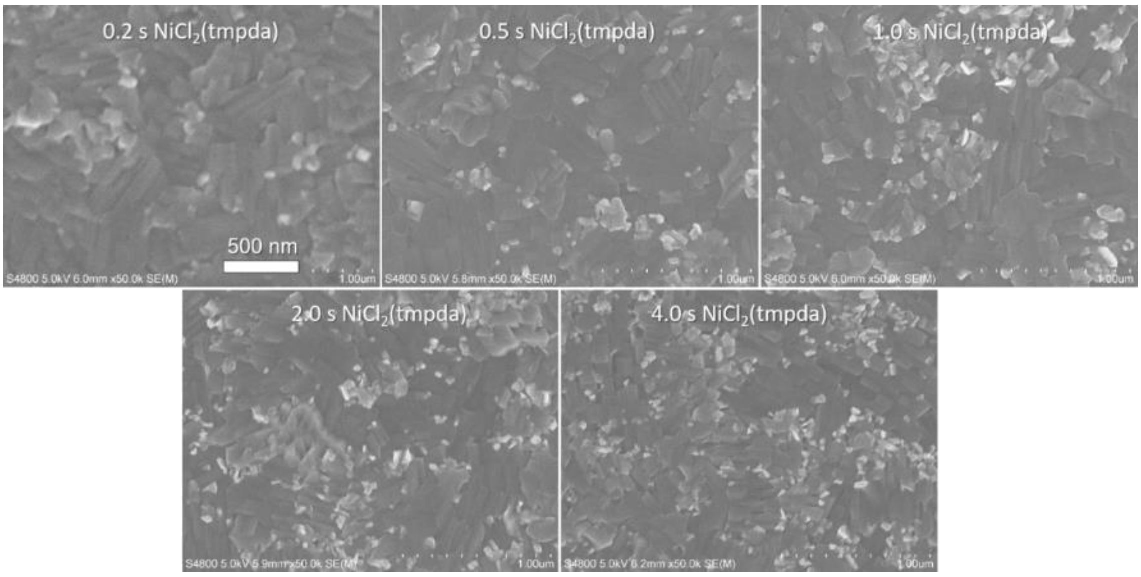

Figure S8. SEM images of films deposited with different $NiCl_2$(tmpda) pulse lengths. The films were grown on silicon at 165 °C using 750 cycles with 4.0 s H_2S pulses and 2.0 s purges.

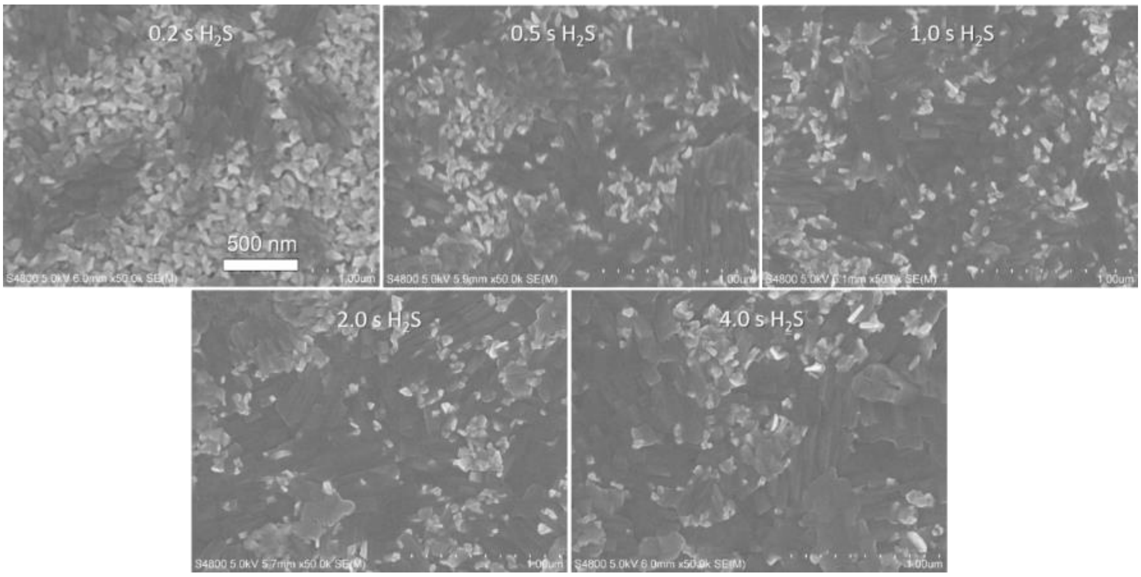

Figure S9. SEM images of films deposited with different H_2S pulse lengths. The films were grown on silicon at 165 °C using 750 cycles with 2.0 s $NiCl_2$(tmpda) pulses and 2.0 s purges.

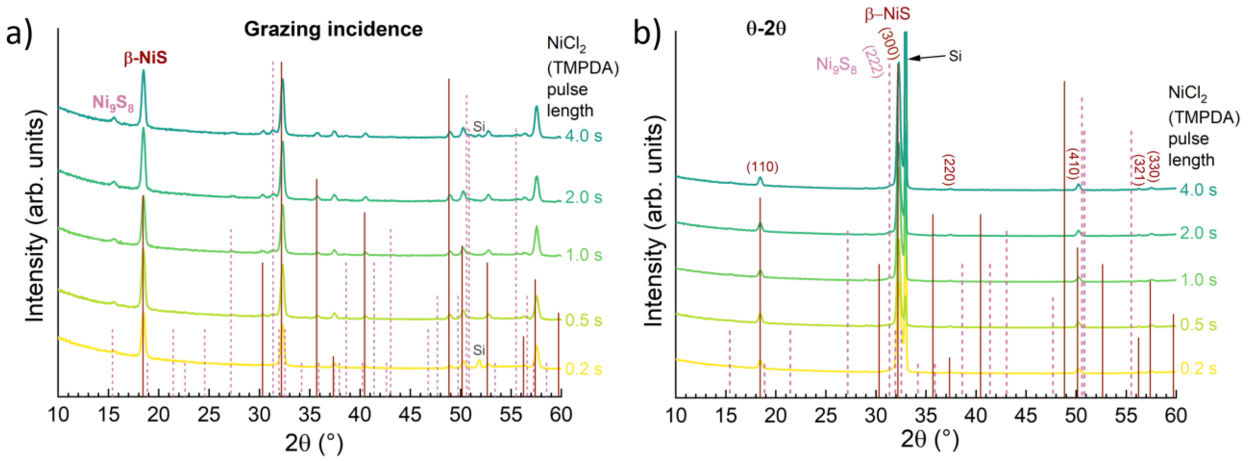

Figure S10. a) Grazing incidence and b) θ-2θ X-ray diffractograms of films deposited with different $NiCl_2$(tmpda) pulse lengths. The films were grown on silicon at 165 °C using 750 cycles with 4.0 s H_2S pulses and 2.0 s purges. The position and height of the lines indicate peak positions and intensities of powder references (PDF 12-41 for β-NiS and 22-1193 for Ni_9S_8). In b), Miller indices are shown for observed peaks only. Peaks originating from the Si substrate (Si) are also indicated.

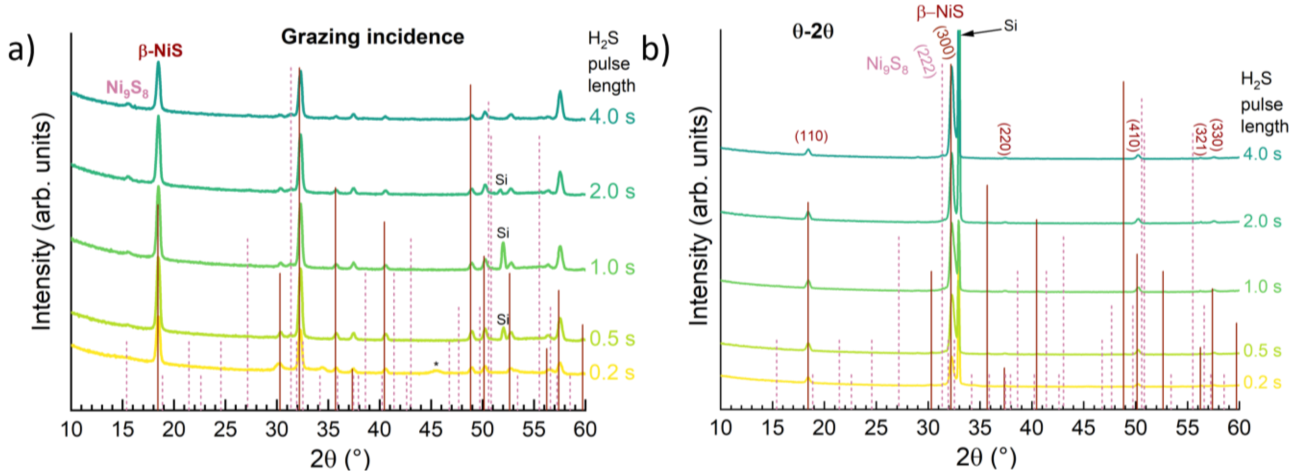

Figure S11. a) Grazing incidence and b) θ-2θ X-ray diffractograms of films deposited with different H_2S pulse lengths. The films were grown on silicon at 165 °C using 750 cycles with 2.0 s $NiCl_2$(tmpda) pulses and 2.0 s purges. The position and height of the lines indicate peak positions and intensities of powder references (PDF 12-41 for β-NiS and 22-1193 for Ni_9S_8). In b), Miller indices are shown for observed peaks only. Peaks originating from the Si substrate (Si) and sample stage of the XRD instrument (asterisk) are also indicated.

.

Section S4. Effect of thickness on film characteristics

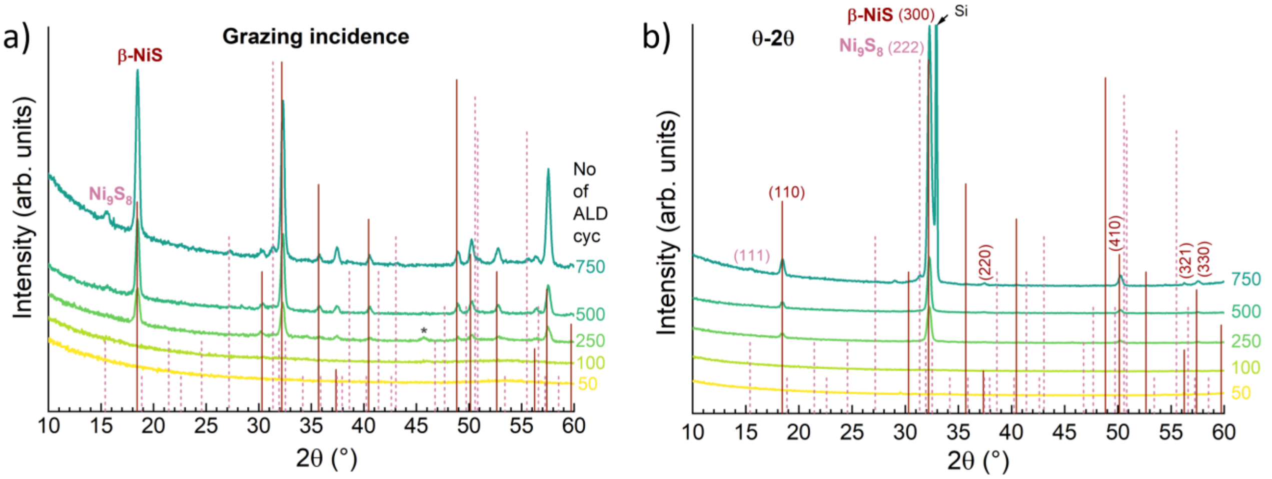

Figure S12. a) Grazing incidence and b) θ-2θ X-ray diffractograms of films deposited on silicon using a varying number of ALD cycles at 165 °C (2.0 s pulses and purges). The position and height of the lines indicate peak positions and intensities of powder references (PDF 12-41 for β-NiS and 22-1193 for Ni_9S_8). In b), Miller indices are shown for observed peaks only. Peaks originating from the Si substrate (Si) and sample stage of the XRD instrument (asterisk) are also indicated.

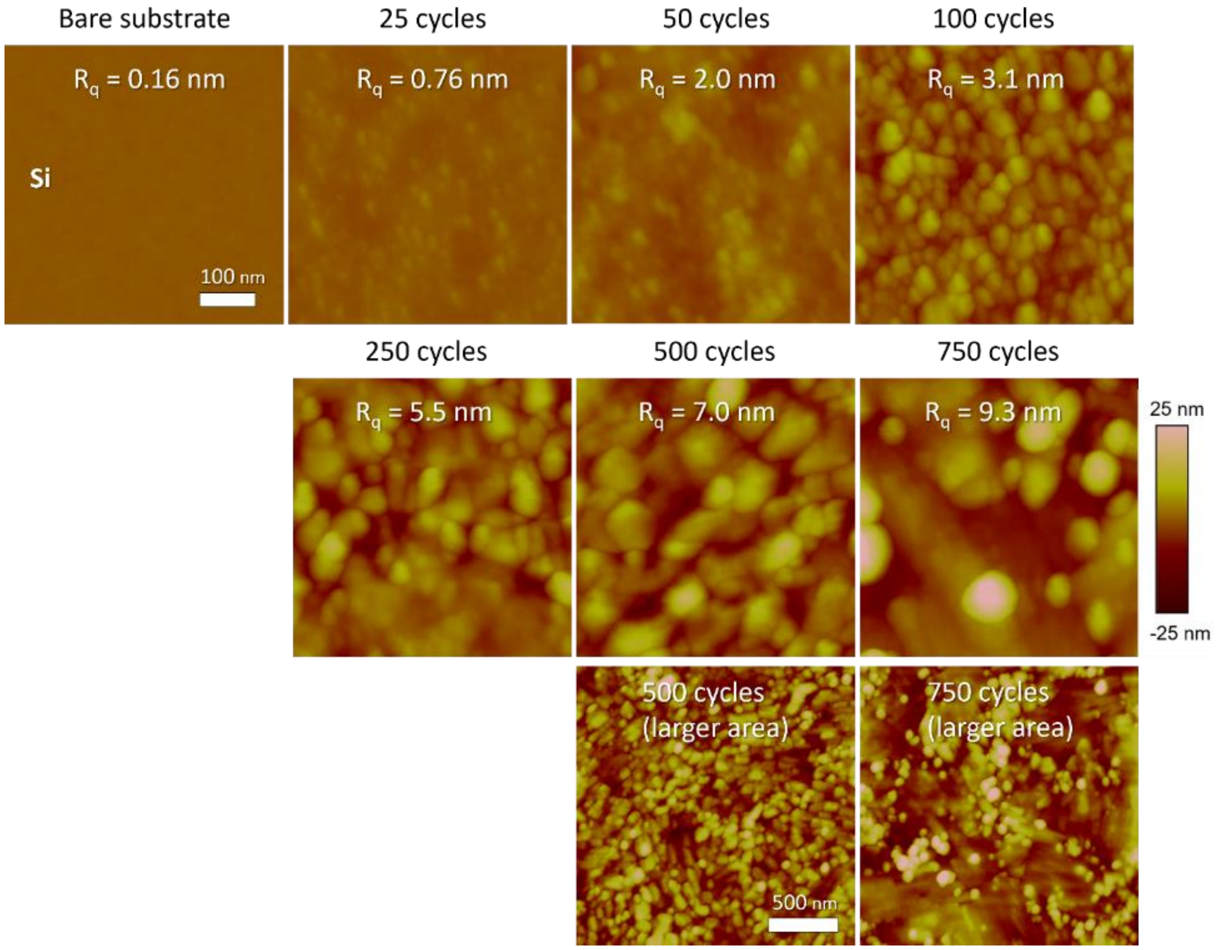

Figure S13. AFM images and roughness values (R_q, average from 3–5 images) of NiS_x films deposited at 165 °C using a varying number of cycles (2.0 s pulses and purges). Image size is 500 × 500 nm^2 except for the 2 × 2 μm^2 images in the bottom row.

Section S5. Comparison to other NiS_x ALD processes

Note S4. Comparison to other NiS_x ALD processes.

Our ALD process is compared to other known processes in terms of deposition temperature, growth rate, crystallinity, impurities, and resistivity (Table S2). The β-NiS and Ni_9S_8 phases observed in our study have also been deposited using some other nickel(II) precursors with H_2S, although amorphous films and crystalline Ni_3S_2 have resulted in other cases. In one study, H_2S plasma was used with $Ni(^tBuAMD)_2$ to achieve the sulfur-rich NiS_2 phase. All of the previous studies have been reported to deposit a single NiS_x phase, while our process has an ability to tune the ratio of β-NiS and Ni_9S_8 phases by varying the deposition temperature (amount of Ni_9S_8 increases with increasing temperature). So far, deposition temperature and post-deposition annealing appear to be the closest available approaches to a general phase control strategy of ALD NiS_x.

The growth rate of our process is higher than that of the Ni_9S_8 process using $Ni(^tBuAMD)_2$ with H_2S (0.15 Å/cycle) and $^tBuSS^tBu$ (0.1–0.3 Å/cycle) and the β-NiS processes using $Ni(thd)_2$ or $Ni(acac)_2$ with H_2S (0.2–0.25 Å/cycle) and close to the β-NiS process using $Ni(dmamb)_2$ and H_2S (0.8 Å/cycle). Interestingly, in two studies the $Ni(dmamb)_2$+H_2S process has been shown to result in Ni_3S_2 films, in one of them with a considerably higher growth rate up to 3.7 Å/cycle. The deposition temperatures of our process are rather typical, although processes operating at both lower and higher temperatures have been reported.

Low impurity levels similar to our $NiCl_2(TMPDA)$+H_2S process have also been reported for the other processes, although none of the other studies have analyzed all of the common impurities including hydrogen. The measured resistivity of our films is comparable to the most conductive ALD NiS_x films reported, although the rough morphology causes some uncertainty in the values. Interestingly, the resistivity of our films is also comparable to ALD Ni_3N films[7] and even many ALD Ni films, which ranges from 20 to as high as 200 µΩcm (Ref.[8] and references therein. The bulk resistivity of nickel is 7 µΩcm[9]).

Table S2. Reported NiS_x ALD processed and selected film characteristics. Conditions cited as non-saturating in the references have been excluded. dl = detection limit.

Precursors	Temperature [°C] (growth rate [Å/cycle])	Crystallinity and phase	Impurities [at-%]	Resistivity [µΩcm] (thickness)	Reference
$NiCl_2(TMPDA)$ + H_2S	165 (0.6) – 225 (0.2)	Mainly β-NiS, some Ni_9S_8	Cl: <1<br>C, O: <0.5<br>N: <0.1<br>H: ~1	40–50 (50 nm)<br>64 (22 nm)<br>270 (6 nm)	This work
$Ni(thd)_2$ + H_2S	175–350 (0.2 @ 175–250 °C)	β-NiS	?	60 (20 nm)	10
$Ni(acac)_2$ + H_2S	200–240 (0.25)	β-NiS	?	165 (26 nm)	11
$Ni(^tBuAMD)_2$ + H_2S	90–200 (0.15)	Ni_9S_8	C: ~1<br>N: <1	?	12
	125–225 (0.2) [a)]	Amorphous $NiS_{0.96}$	?	?	13
$Ni(^tBuAMD)_2$ + $^tBuSS^tBu$	180 (0.1) – 220 (0.3)	Ni_9S_8	C: 1.3<br>N: <0.5	?	14
$Ni(^tBuAMD)_2$ + H_2S plasma	80 (0.8) – 300 (1.2)	NiS_2	N, C, O: <1	?	15
$Ni(dmamb)_2$ + H_2S	250 (0.75)	rhombohedral Ni_3S_2	?	38 (150 nm)	16
	80–160 (0.8)	β-NiS	?	370 (? nm)	17
	90 (1.2) – 190 (3.7)	Ni_3S_2 (mix of two phases)	O, C: <dl	65 (100 nm)	18

a) Steady-state growth rate. The growth rate was higher, 0.5 Å/cycle for the first 50 cycles on SiO_2.

Section S6. HTXRD measurements and post-characterization

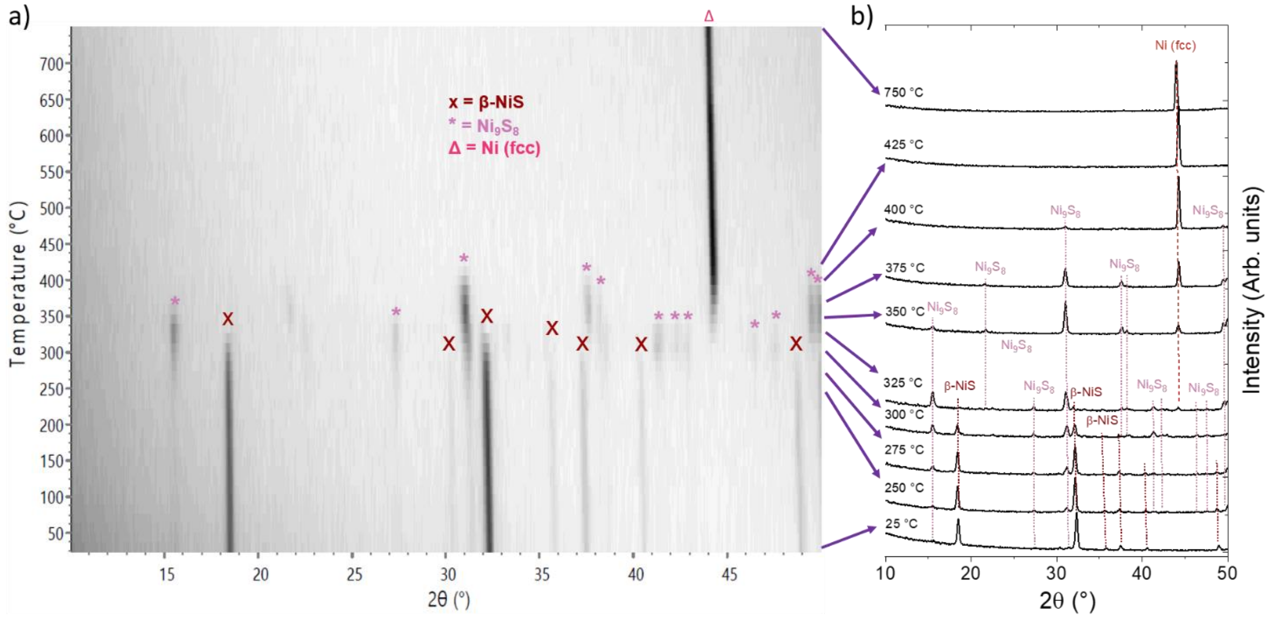

Figure S14. a) High-temperature grazing incidence X-ray diffractograms measured in a forming gas atmosphere (10% H_2 / 90% N_2, 1 atm) from 25 to 750 °C (measured at 25 °C intervals), where light and dark represent low and high intensities in a square-root scale. b) Selected diffractograms measured at different temperatures. The studied film was deposited on silicon at 165 °C using 750 cycles (2.0 s pulses and purges).

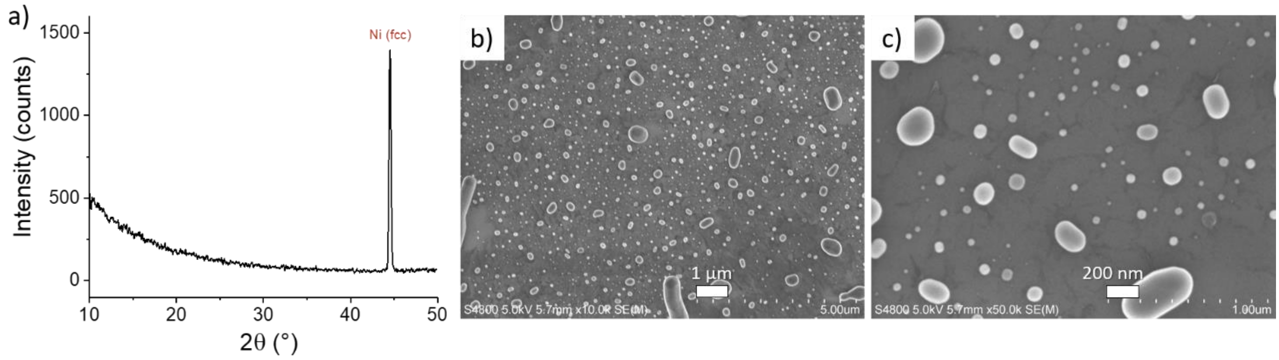

Figure S15. a) Grazing incidence X-ray diffractogram (at 25 °C) and b,c) SEM images after HTXRD measurement in a forming gas atmosphere (10% H_2 / 90% N_2, 1 atm) up to 750 °C.

Note S5. **Annealing in N_2 (inert) atmosphere.**

Under an inert N_2 atmosphere, the initially present β-NiS phase was retained up to 400 °C and Ni_9S_8 up to 475 °C (Figure S16). At higher temperatures, orthorhombic α-Ni_7S_6 – a known high-temperature phase (PDF 25-583)[19] – formed and was present as the only phase up to 575 °C. Thus, N_2 annealing at approximately 400 or 500 °C could possibly be used to convert the films to Ni_9S_8 and α-Ni_7S_6, respectively. Formation of α-Ni_7S_6 from β-NiS or Ni_9S_8 requires loss of some sulfur, which is easily evaporated at these temperatures, or formation of a sulfur rich phase, such as Ni_4S_3 or NiS_2 (not detected). Following a possible intermediate phase forming at 575 °C that could not be identified, NiSi formed above 600 °C assigned to a reaction with the Si substrate. However, sulfur was not completely lost from the films, as rhombohedral Ni_3S_2 (heazlewoodite, PDF 44-1418) formed at ~600 °C and was present when the film was cooled down from 750 °C to room temperature (Figure S17). The three unidentified peaks at 650–750 °C may thus result from some nickel sulfide phase, although it could not be definitively identified. The observed transformation temperatures to α-Ni_7S_6 and Ni_9S_8 are in line with previous reports on bulk material, as is the formation of Ni_3S_2 upon

decomposition of α-Ni_7S_6.[19] Thus, besides the aforementioned Ni_9S_8 and α-Ni_7S_6, the deposited (mostly β-NiS) film could likely be converted to Ni_3S_2 at 600 °C or above if a substrate other than silicon was used or a diffusion barrier was deposited between Si and the NiS_x films.

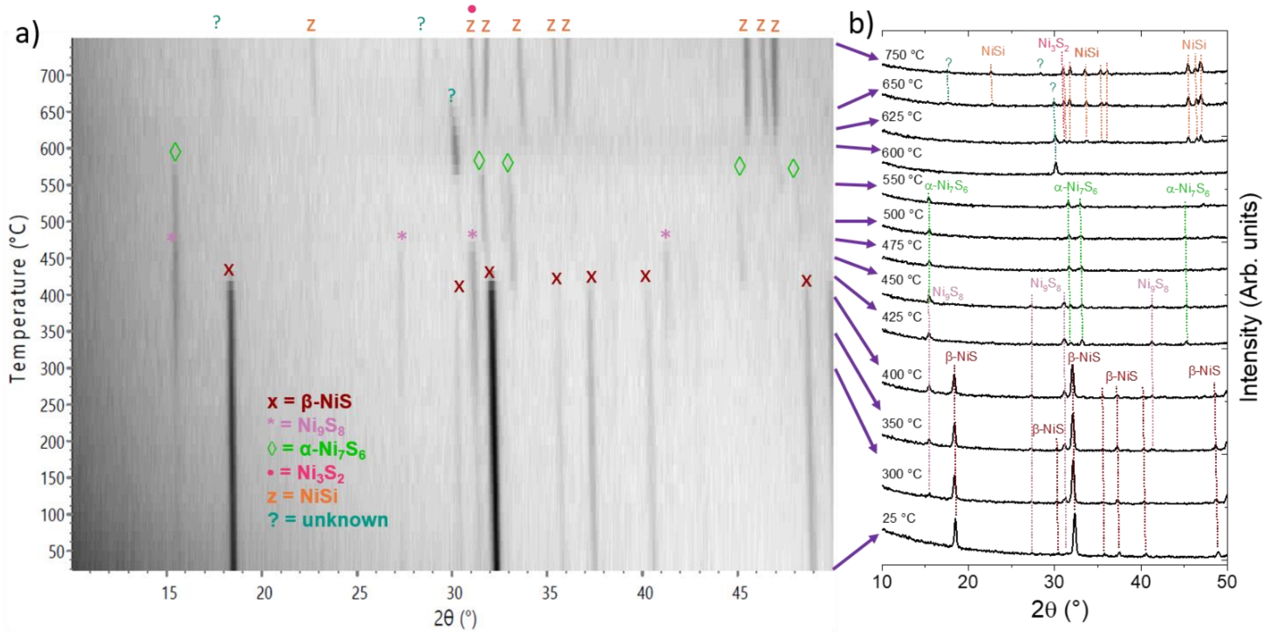

Figure S16. a) High-temperature grazing incidence X-ray diffractograms measured in a N_2 atmosphere (1 atm) from 25 to 750 °C (measured at 25 °C intervals), where light and dark represent low and high intensities in a square-root scale. b) Selected diffractograms measured at different temperatures. The studied film was deposited on silicon at 165 °C using 750 cycles.

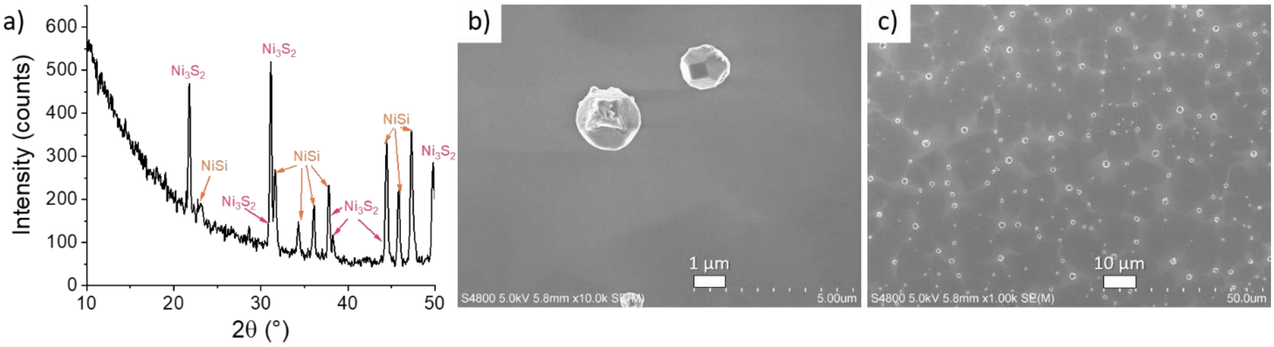

Figure S17. a) Grazing incidence X-ray diffractogram (at 25 °C) and b,c) SEM images after HTXRD measurement in a N_2 atmosphere (1 atm) up to 750 °C.

Note S6. Annealing in vacuum (inert) atmosphere.

β-NiS and Ni_9S_8 phases disappeared at 350 and 375 °C and α-Ni_7S_6 was observed between 375 and 475 °C with NiSi starting to form at 450 °C (Figure S18). After heating up to 750 °C and cooling back to room temperature, Ni_3S_2 was observed in addition to NiSi, although the Ni_3S_2/NiSi intensity ratio was much lower compared to the N_2 atmosphere (Figure S19). Overall, similar phase transitions were observed for vacuum and N_2, but they occurred at a lower temperature in case of a vacuum atmosphere.

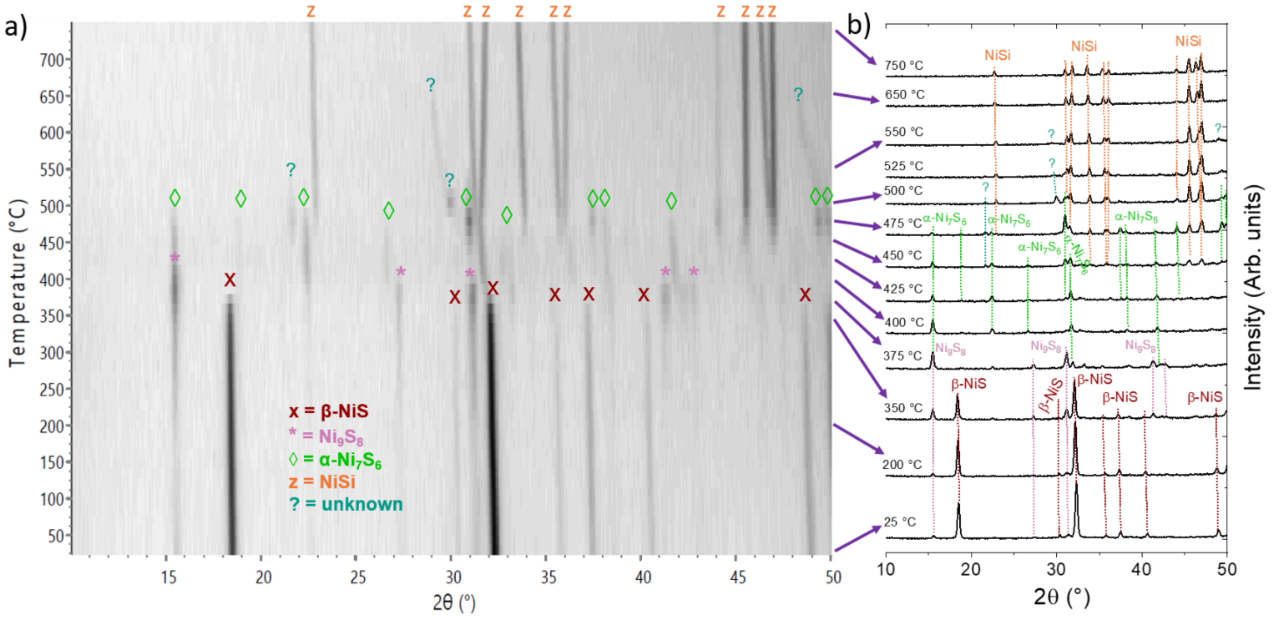

Figure S18. a) High-temperature grazing incidence X-ray diffractograms measured in vacuum (approximately 10^{-5} mbar) from 25 to 750 °C (measured at 25 °C intervals), where light and dark represent low and high intensities in a square-root scale. b) Selected diffractograms measured at different temperatures. The studied film was deposited on silicon at 165 °C using 750 cycles.

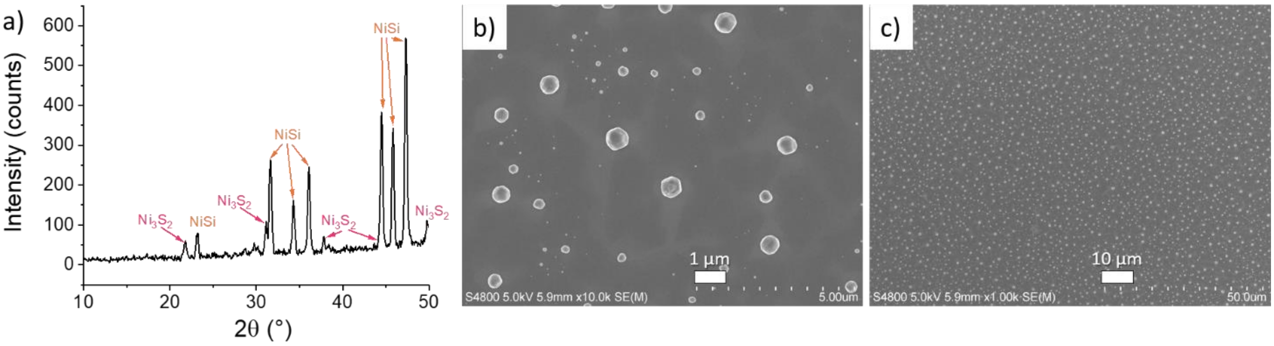

Figure S19. a) Grazing incidence X-ray diffractogram (at 25 °C) and b,c) SEM images after HTXRD measurement in vacuum (approximately 10^{-5} mbar) up to 750 °C.

Note S7. Annealing in ambient air and O_2 (oxidizing) atmospheres

In ambient air, the first changes were observed at approximately 250 °C, where Ni_9S_8 disappeared. The other initially present NiS_x phase, β-NiS, was present until 300 °C. At this temperature, a minor Ni_3S_4 (polydymite, PDF 47-1739) component started to form (Figure S20). The formation of species richer in sulfur than the original Ni_9S_8 or β-NiS also requires a sulfur-poor phase to form. As no other crystalline phases were seen, this sulfur-poor phase may be amorphous, likely NiO.[20] Hexagonal α-NiS (PDF 2-1280), a known high-temperature form of NiS, began to form at 325 °C. It is interesting that in our study the α-NiS phase was only observed in oxidizing atmosphere and not in inert atmosphere. At 375–450 °C, the film seemed to be mostly composed of α-NiS, at least in terms of crystalline phases. However, broad peaks originating from cubic NiO (bunsenite, PDF 47-1049) also started to emerge at these temperatures, likely suggesting crystallization of the amorphous oxide phase. The α-NiS phase disappeared by 450 °C and a $NiSO_4$ phase (PDF 13-435), a known oxidation product of NiS,[20] was observed between 500 and 550 °C in addition to NiO. Following disappearance of $NiSO_4$, no signs of sulfur-containing phases were seen, suggesting that $NiSO_4$ either decomposed directly to NiO and SO_x (and possibly O_2), or that the Ni_3S_2 decomposition product suggested in literature[20] quickly oxidized to NiO also producing SO_x. Thereafter, the crystallinity of NiO improved upon an increase of temperature up to 750 °C as is evident from the increased intensity and decreased width of reflections. After

annealing, well crystallized but rough NiO films were obtained (Figure S21). When pure O_2 was used instead of ambient air, similar phase transitions were observed, although at temperatures 25–50 °C lower compared to air (Figures S22,S23).

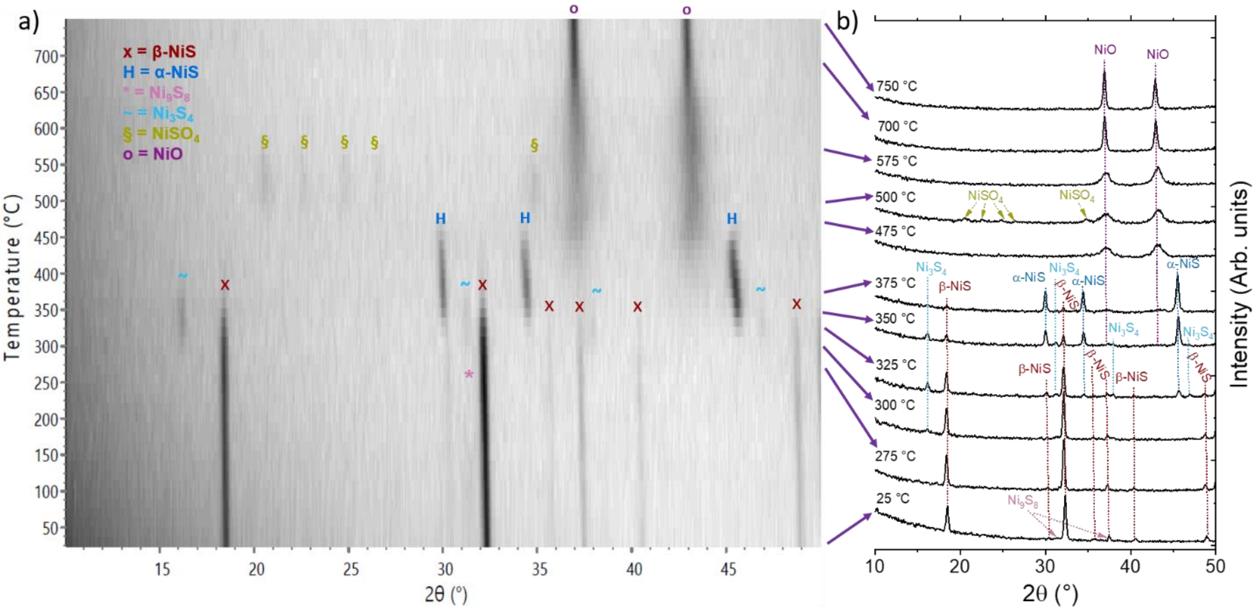

Figure S20. a) High-temperature grazing incidence X-ray diffractograms measured in ambient air from 25 to 750 °C (measured at 25 °C intervals), where light and dark represent low and high intensities in a square-root scale. b) Selected diffractograms measured at different temperatures. The studied film was deposited on silicon at 165 °C using 750 cycles.

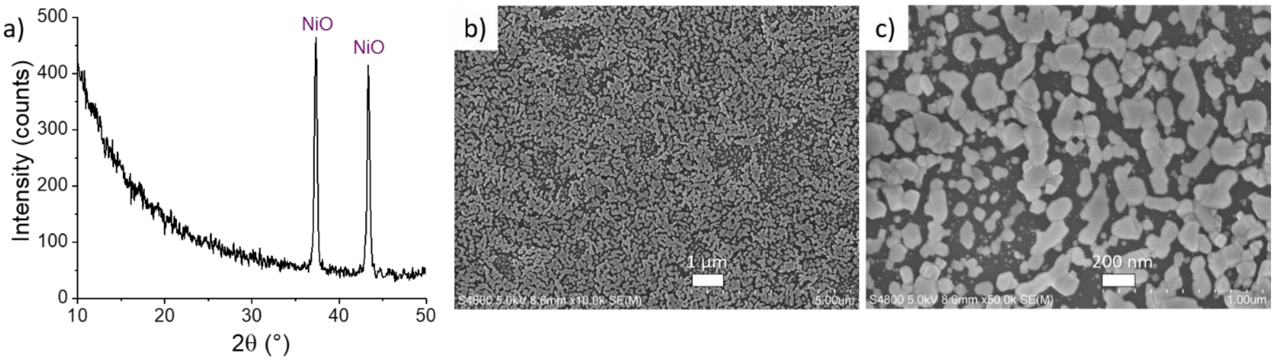

Figure S21. a) Grazing incidence X-ray diffractogram (at 25 °C) and b,c) SEM images after HTXRD measurement in air up to 750 °C.

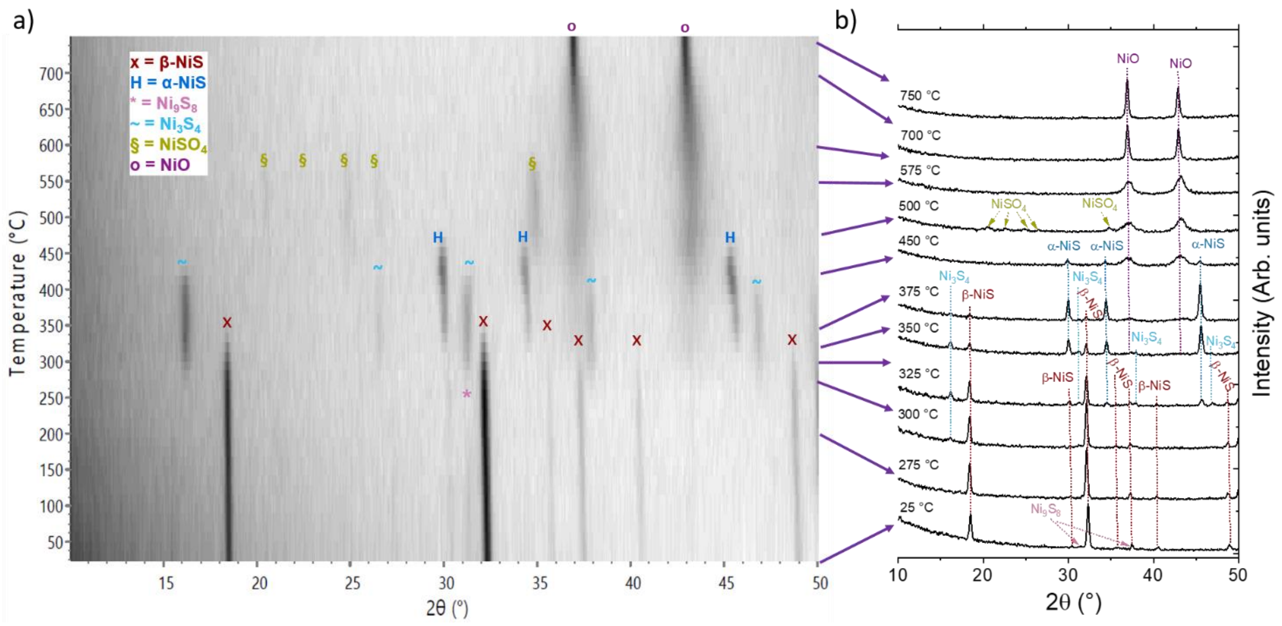

Figure S22. High-temperature grazing incidence X-ray diffractograms during heating in an O_2 atmosphere (1 atm) from 25 to 750 °C (measured at 25 °C intervals), where light and dark represent low and high intensities, respectively. b) Selected diffractograms measured at different temperatures. The studied film was deposited on silicon at 165 °C using 750 cycles.

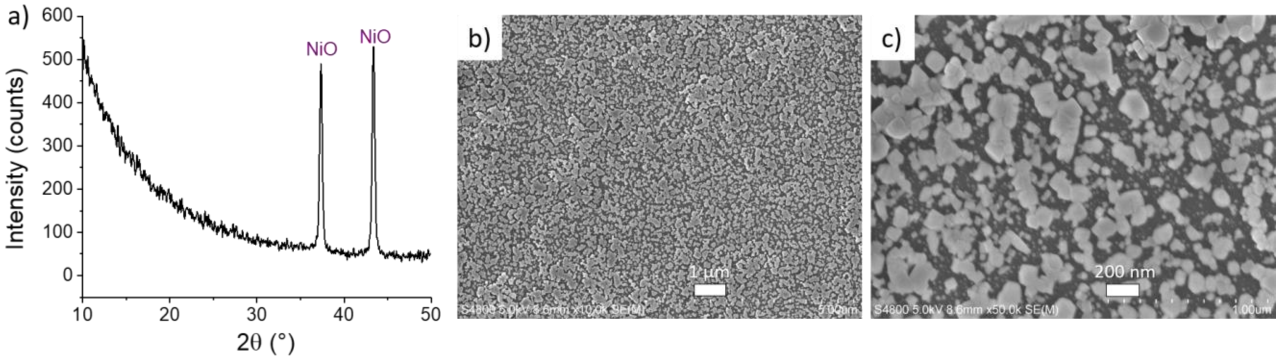

Figure S23. a) Grazing incidence X-ray diffractogram (at 25 °C) and b,c) SEM images after HTXRD measurement in O_2 up to 750 °C.

Section S7. Characterization of films deposited on FTO vs Si

Note S8. Comparison of NiS_x growth and properties on FTO and Si.

As ALD is a surface-controlled technique, the substrate has a strong effect on film growth. Compared to the silicon substrates used throughout the majority of the study, on the FTO substrates used for electrochemical measurements the film growth was initially slower (up to 250 ALD cycles, Figure S24). The lower thickness on FTO suggests a longer nucleation delay on FTO attributed to a lower amount and/or lower reactivity of surface sites. With an increasing number of ALD cycles, however, the thickness on FTO rapidly increased and appeared to exceed that on Si. The subsequent faster growth may be due to the much larger roughness and consequently surface area of FTO compared to Si (Figure S26). Using AFM, the roughness and surface area of FTO were found to be 47±2 nm (root-mean-square) and 1.45±0.01 times the geometric area (average±standard deviation of three 5×5 µm images). The EDS measurements did not account for the substrate roughness and/or surface area. Thus, the thickness values only represent an approximation. The differences in NiS_x morphology on different substrates can also affect growth rate (Figure S26). Regardless, on both the Si and FTO substrates the films were found to consist mainly of β-NiS (Figure S25).

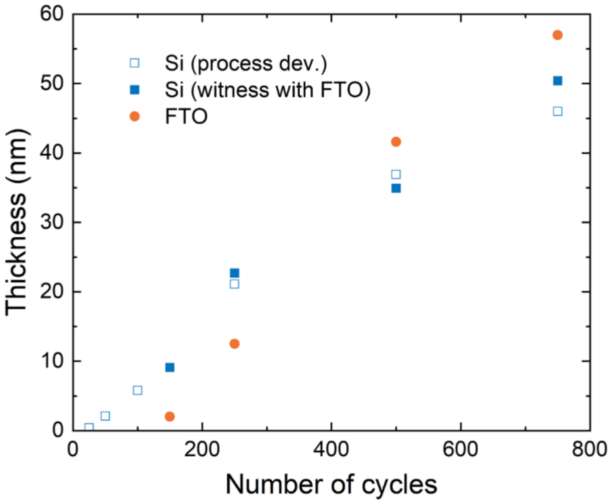

Figure S24. **Apparent thickness on Si and FTO substrates measured by EDS**. For Si, data from process development experiments (Si and soda lime glass substrates facing each other) and FTO depositions (Si facing FTO substrates held in place by an aluminum adapter) are shown. The depositions were done using 2.0 s $NiCl_2$(tmeda) and H_2S pulses and purges at 165 °C.

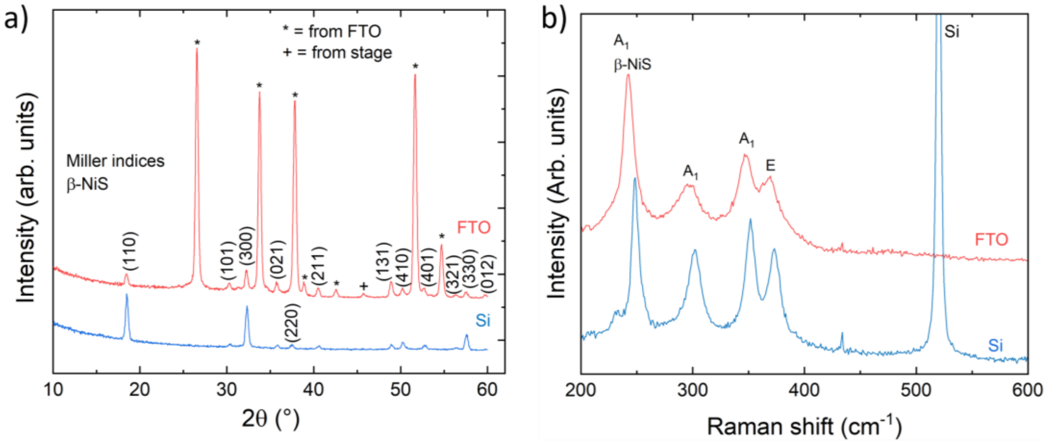

Figure S25. **Crystallinity on Si and FTO substrates** measured by a) gracing incidence XRD (asterisk marks FTO reflections, Miller indices refer to β-NiS) and b) Raman spectroscopy (symmetry assignments of β-NiS according to Ref.[1] . The films were deposited at 165 °C using 500 cycles consisting of 2.0 s $NiCl_2$(tmeda) and H_2S pulses separated by 2.0 s N_2 purges.

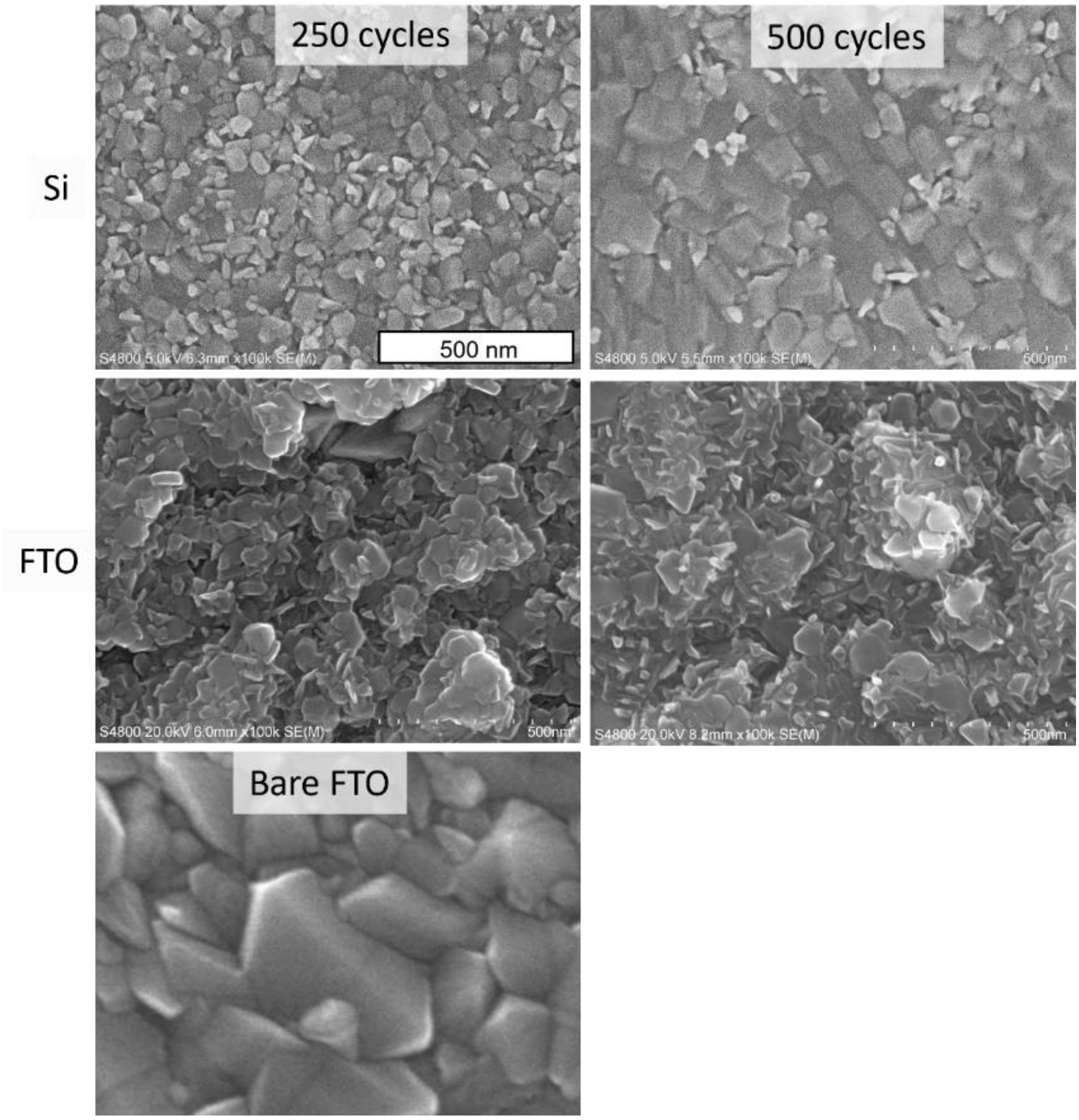

Figure S26. Morphology on Si and FTO substrates (SEM images). The films were deposited at 165 °C using 250 and 500 cycles consisting of 2.0 s $NiCl_2$(tmeda) and H_2S pulses separated by 2.0 s N_2 purges. For comparison, a bare FTO substrate is also shown (bare Si substrate appeared featureless). All images are in the same scale.

Section S8. NiS Pourbaix diagrams

Note S9. Pourbaix diagrams.

Pourbaix diagrams predict the thermodynamically stable species in aqueous conditions of a given pH and potential. It is worth noting that reactions from one species to another usually involve an activation energy, which can lead to a thermodynamically unstable species being observed in practice. In addition, the accuracy of Pourbaix diagrams depends on the accuracy of available thermodynamic data, either experimental (Figures S28) or calculated (Figure S27). Thus, they best serve as a semi-quantitative guide, as used here to argue for the limited stability of NiS under water splitting conditions.

Under acidic HER conditions, soluble Ni^{2+} species are predicted to be stable, although at negative enough potentials Ni metal can become stable. Under acidic OER conditions, dissolution as Ni^{2+} is predicted. Under alkaline conditions, Ni dissolution is less likely, but transformation of NiS to Ni metal (HER) or NiOOH (OER) is predicted.

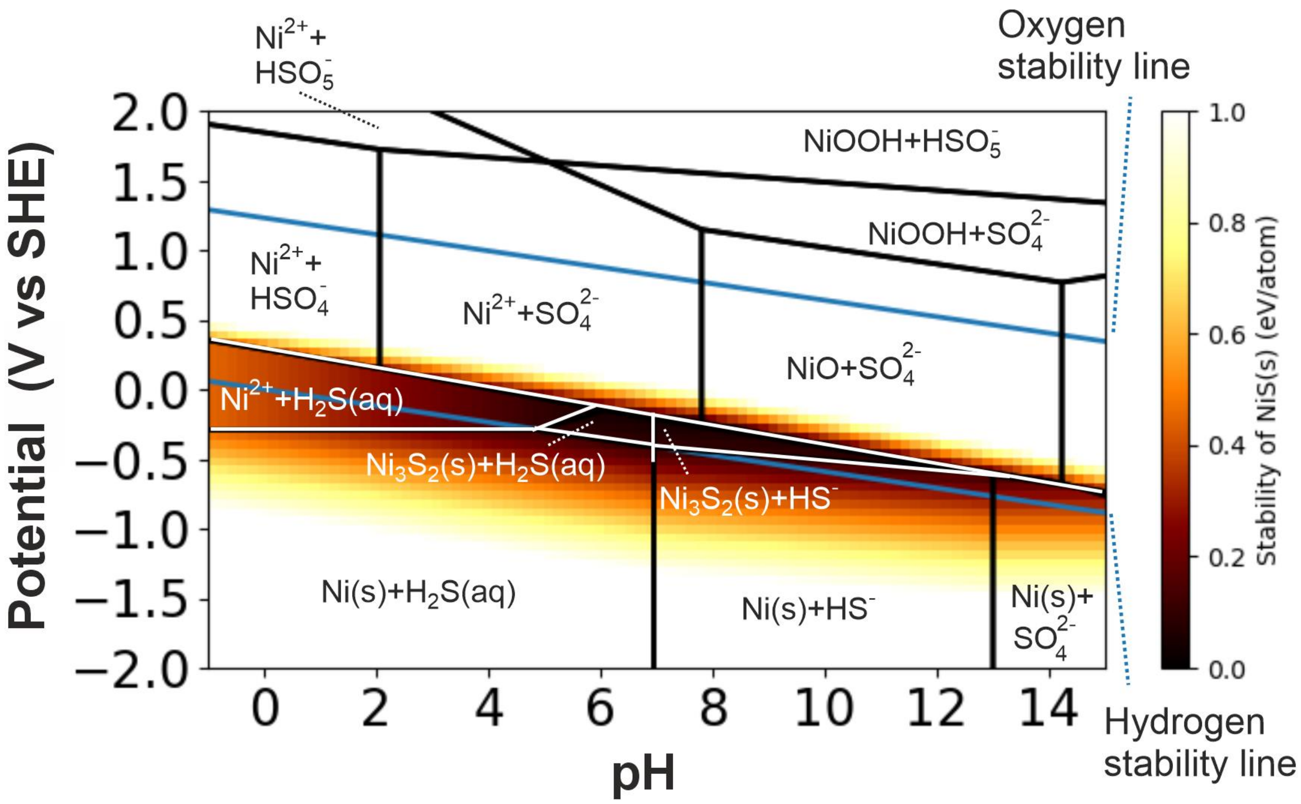

Figure S27. Pourbaix diagram (both Ni and S species) generated for aqueous Ni-S system using thermodynamic data calculated by the Materials Project (composition NiS, ion concentrations 10^{-3} M).[21–24] The overlaid coloring represents the Gibbs free energy of β-NiS (mp-1547), where black (<0 eV/atom) represents the thermodynamically stable region and lighter colors regions where there is a stronger driving force for decomposition of NiS. Solid species are denoted with (s) and neutral aqueous species (aq). All charged species are aqueous. SHE = standard hydrogen electrode.

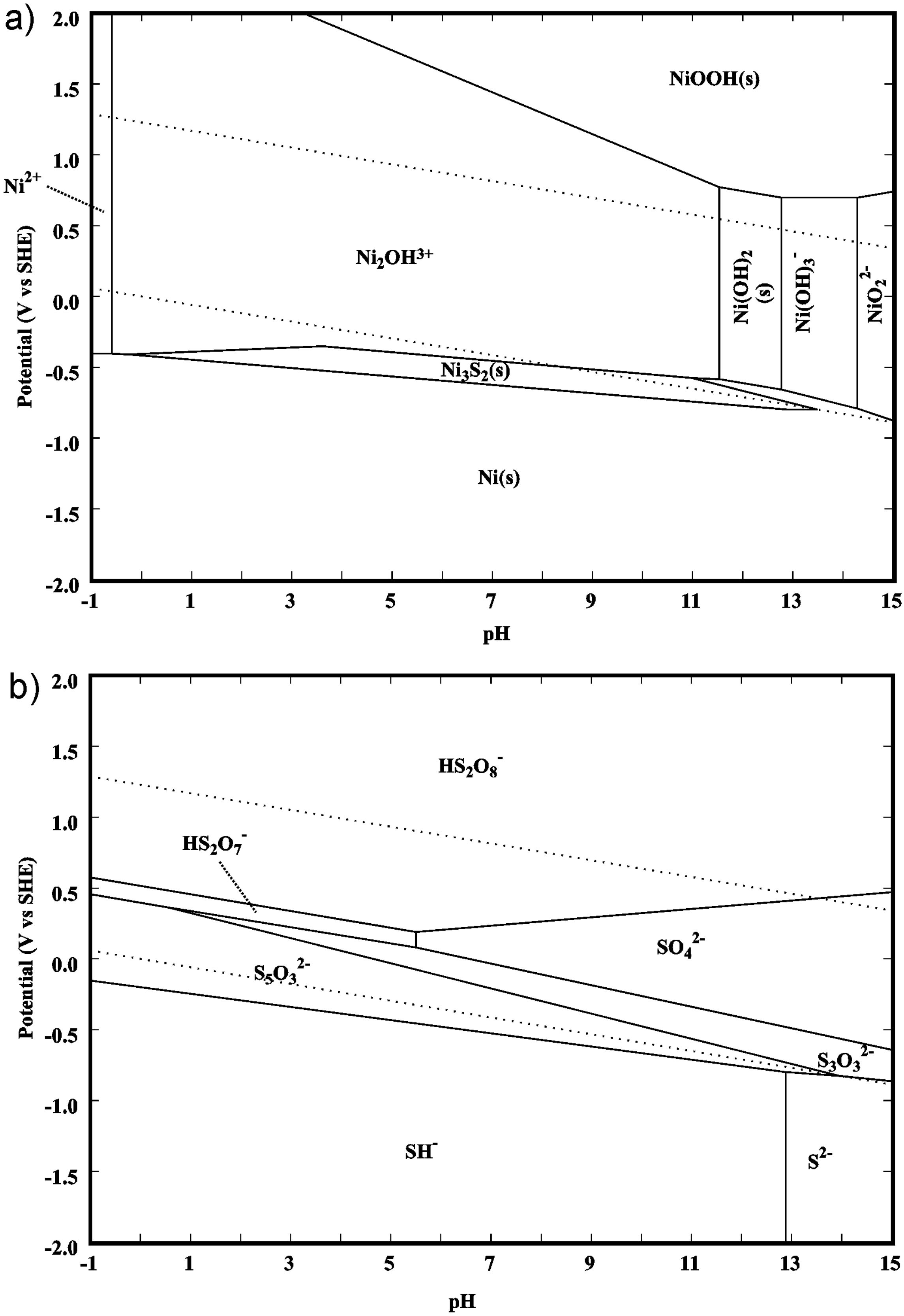

Figure S28. Pourbaix diagram generated for aqueous Ni-S system based on experimental data shown for a) Ni and b) S species. The diagrams were drawn using HSC Chemistry (Version 7.0, Outotec Research, Finland) using an ion concentration of 10^{-6} M. Data for NiH_x species were excluded due to their reported instability and high barrier to formation.[25]

Section S9. Electrochemical experiments and before/after characterization

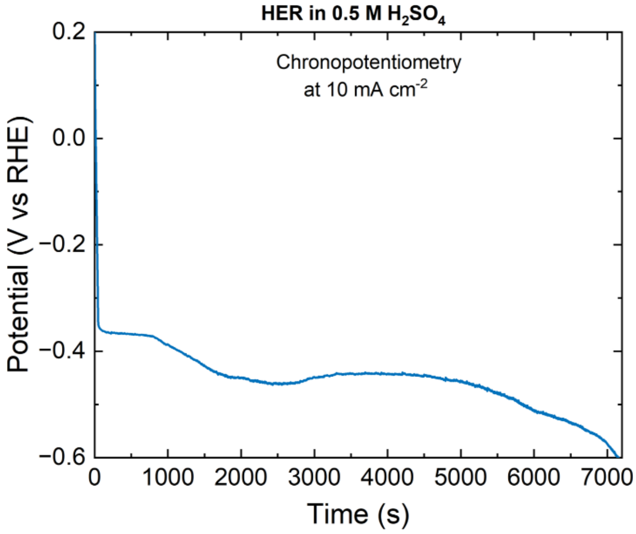

Figure S29. Chronopotentiometry of a NiS_x film (deposited at 165 °C, 500 ALD cycles) on FTO at 10 mA/cm^2 in 0.5 M H_2SO_4 showing a large, over 200 mV increase in required overpotential during 2 hours.

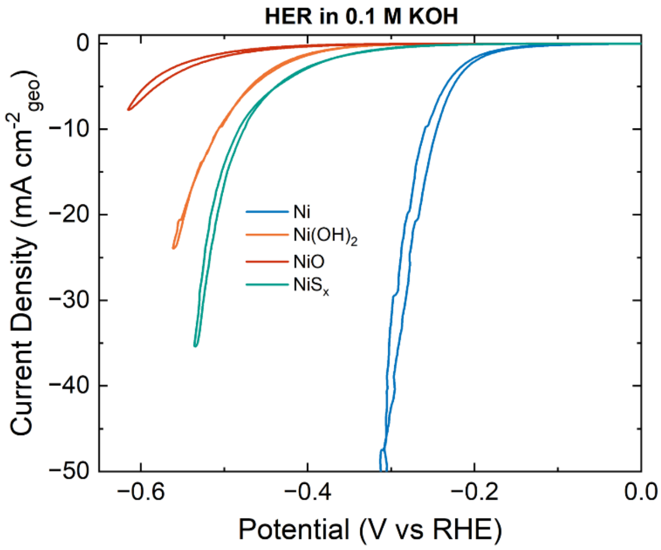

Figure S30. Comparison of different Ni-based materials for HER in 0.1 M KOH. The 20th CV is shown. The reference materials were prepared at a similar, 10–20 nm thickness on FTO. NiO was prepared by ALD ($NiCp_2$ and O_3 at 250 °C), Ni by electron-beam evaporation at room temperature, and $Ni(OH)_2$ by electrodeposition (cathodically from a 0.1 M $Ni(NO_3)_2$ solution).

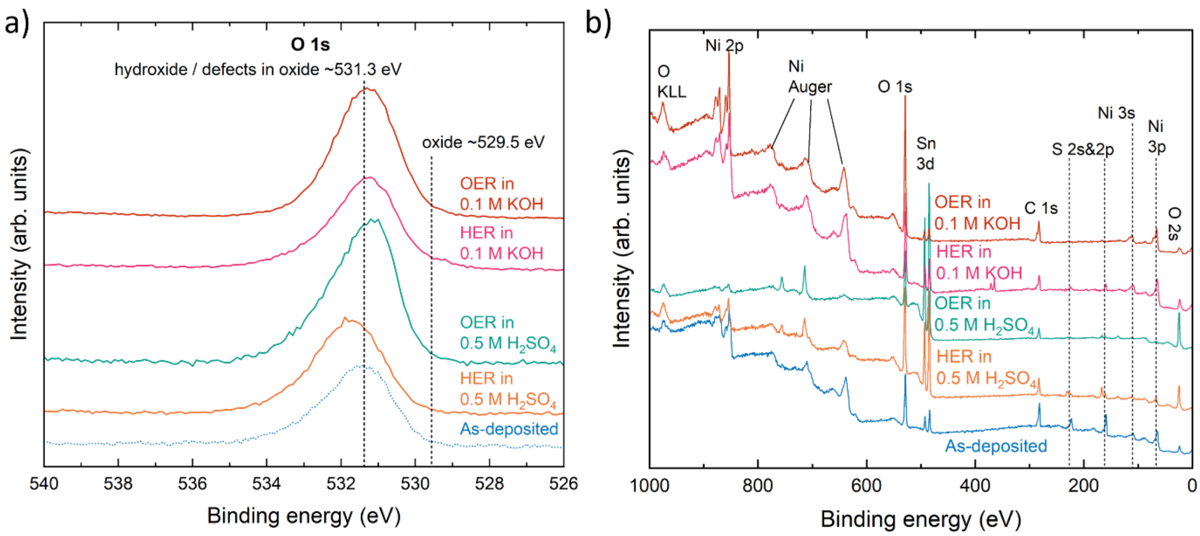

Figure S31. X-ray photoelectron spectra of NiS_x film as deposited and after electrochemical CV experiments. a) O 1s region and b) survey scans supporting the spectra in Figure 6.

Section S10. References

1 F. Guillaume, S. Huang, K. D. M. Harris, M. Couzi and D. Talaga, Optical phonons in millerite (NiS) from single-crystal polarized Raman spectroscopy, *J. Raman Spectrosc.*, 2008, **39**, 1419–1422.

2 Z. Cheng, H. Abernathy and M. Liu, Raman spectroscopy of nickel sulfide Ni_3S_2, *J. Phys. Chem. C*, 2007, **111**, 17997–18000.

3 D. W. Bishop, P. S. Thomas and A. S. Ray, Micro Raman characterization of nickel sulfide inclusions in toughened glass, *Mater. Res. Bull.*, 2000, **35**, 1123–1128.

4 A. R. Neale, Y. Jin, J. Ouyang, S. Hughes, D. Hesp, V. Dhanak, G. Dearden, S. Edwardson and L. J. Hardwick, Electrochemical performance of laser micro-structured nickel oxyhydroxide cathodes, *J. Power Sources*, 2014, **271**, 42–47.

5 S. L. Yang, H. Bin Yao, M. R. Gao and S. H. Yu, Monodisperse cubic pyrite NiS_2 dodecahedrons and microspheres synthesized by a solvothermal process in a mixed solvent: Thermal stability and magnetic properties, *CrystEngComm*, 2009, **11**, 1383–1390.

6 J. Deng, Q. Gong, H. Ye, K. Feng, J. Zhou, C. Zha, J. Wu, J. Chen, J. Zhong and Y. Li, Rational Synthesis and Assembly of Ni_3S_4 Nanorods for Enhanced Electrochemical Sodium-Ion Storage, *ACS Nano*, 2018, **12**, 1829–1836.

7 K. Väyrynen, T. Hatanpää, M. Mattinen, M. J. Heikkilä, K. Mizohata, J. Räisänen, J. Link, R. Stern, M. Ritala and M. Leskelä, Atomic Layer Deposition of Nickel Nitride Thin Films using $NiCl_2$(TMPDA) and Tert-Butylhydrazine as Precursors, *Phys. Status Solidi*, 2019, **216**, 1900058.

8 K. Väyrynen, PhD thesis, University of Helsinki, 2019, http://hdl.handle.net/10138/305438.

9 *CRC Handbook of Chemistry and Physics*, http://www.hbcpnetbase.com/, (accessed May 2023).

10 N. Mahuli and S. K. Sarkar, Atomic layer deposition of NiS and its application as cathode material in dye sensitized solar cell, *J. Vac. Sci. Technol. A*, 2016, **34**, 01A142.

11 R. Singh and M. M. Ayyub, Atomic Layer Deposition of Crystalline β-NiS for Superior Sensing in Thin-Film Non-Enzymatic Electrochemical Glucose Sensors, *ACS Appl. Electron. Mater.*, 2021, **3**, 1912–1919.

12 H. Li, Y. Shao, Y. Su, Y. Gao and X. Wang, Vapor-Phase Atomic Layer Deposition of Nickel Sulfide and Its Application for Efficient Oxygen-Evolution Electrocatalysis, *Chem. Mater.*, 2016, **28**, 1155–1164.

13 Y. Çimen, A. W. Peters, J. R. Avila, W. L. Hoffeditz, S. Goswami, O. K. Farha and J. T. Hupp, Atomic Layer Deposition of Ultrathin Nickel Sulfide Films and Preliminary Assessment of Their Performance as Hydrogen Evolution Catalysts, *Langmuir*, 2016, **32**, 12005–12012.

14 H. Li, R. Zhao, J. Zhu, Z. Guo, W. Xiong and X. Wang, An Organosulfur Precursor for Atomic Layer Deposition of High-Quality Metal Sulfide Films, *Chem. Mater.*, 2020, **32**, 8885–8894.

15 Z. Guo and X. Wang, Atomic Layer Deposition of the Metal Pyrites FeS_2, CoS_2, and NiS_2, *Angew. Chemie Int. Ed.*, 2018, **57**, 5898–5902.

16 T. A. Ho, C. Bae, H. Nam, E. Kim, S. Y. Lee, J. H. Park and H. Shin, Metallic Ni_3S_2 Film Grown by Atomic Layer Deposition as an Efficient and Stable Electrocatalyst for Overall Water Splitting, *ACS Appl. Mater. Interfaces*, 2018, **10**, 12807–12815.

17 M. H. Ko, B. Shong and J. H. Hwang, Low temperature atomic layer deposition of nickel sulfide and nickel oxide thin films using $Ni(dmamb)_2$ as Ni precursor, *Ceram. Int.*, 2018, **44**, 16342–16351.

18 S. Cho, H. Kim and M. Mo Sung, Rapid growth of NiS_x by atomic layer infiltration and its application as an efficient counter electrode for dye-sensitized solar cells, *J. Ind. Eng. Chem.*, 2019, **77**, 470–476.

19 S. Stølen, H. Fjellvåg, F. Grønvold, H. Seim and E. F. Westrum, *J. Chem. Thermodyn.*, 1994, 26, 987–1000.

20 J. G. Dunn and C. E. Kelly, A TG/DTA/MS study of the oxidation of nickel sulphide, *J. Therm. Anal.*, 1977, **12**, 43–52.

21 A. Jain, S. P. Ong, G. Hautier, W. Chen, W. D. Richards, S. Dacek, S. Cholia, D. Gunter, D. Skinner, G. Ceder and K. A. Persson, Commentary: The Materials Project : A materials genome approach to accelerating materials innovation, *APL Mater.*, 2013, **1**, 011002.

22 K. A. Persson, B. Waldwick, P. Lazic and G. Ceder, Prediction of solid-aqueous equilibria: Scheme to combine first-principles calculations of solids with experimental aqueous states, *Phys. Rev. B*, 2012,

85, 235438.
23 A. K. Singh, L. Zhou, A. Shinde, S. K. Suram, J. H. Montoya, D. Winston, J. M. Gregoire and K. A. Persson, Electrochemical Stability of Metastable Materials, *Chem. Mater.*, 2017, **29**, 10159–10167.
24 A. M. Patel, J. K. Nørskov, K. A. Persson and J. H. Montoya, Efficient Pourbaix diagrams of many-element compounds, *Phys. Chem. Chem. Phys.*, 2019, **21**, 25323–25327.
25 B. Baranowski and S. M. Filipek, 45 Years of nickel hydride — History and perspectives, *J. Alloys Compd.*, 2005, **406**, 2–6.
